# Migrasomes in Ischemic Stroke: Molecular Landscape and Pathophysiological Impact

**DOI:** 10.1002/advs.202520608

**Published:** 2025-12-03

**Authors:** Huifen Zhou, Yingying Zhang, Peng Zhou, Haofang Wan, Lingling Li, Bin Xu, Yuping Wan, Yingming Song, Yi Kang, Hongbo Zhang, Min Shi, Qun Hou, Jiehong Yang, Chen Ding, Wei Fu, Buchang Zhao, Haitong Wan

**Affiliations:** ^1^ School of Basic Medical Sciences Zhejiang Chinese Medical University No. 548 Binwen Road Hangzhou Zhejiang 310053 P. R. China; ^2^ Zhejiang Key Laboratory of Chinese Medicine for Cardiovascular and Cerebrovascular Disease No. 548 Binwen Road Hangzhou Zhejiang 310053 P. R. China; ^3^ Academy of Chinese Medical Sciences Zhejiang Chinese Medical University No. 548 Binwen Road Hangzhou Zhejiang 310053 P. R. China; ^4^ State Key Laboratory of Genetic Engineering and Collaborative Innovation Center for Genetics and Development School of Life Sciences Human Phenome Institute Fudan University No. 220 Handan Road Shanghai 200433 P. R. China; ^5^ Department of Neurology The Second Affiliated Hospital of Zhejiang Chinese Medical University No.318 owang Road Hangzhou Zhejiang 310000 P. R. China; ^6^ Department of Acupuncture Jingzhou Hospital of Traditional Chinese Medicine No.172, East Jiangjin Road Jingzhou Hubei 434000 P. R. China; ^7^ Department of Encephalopathy Luohe Hospital of Traditional Chinese Medicine No.649, South Traffic Road Luohe Henan 462000 P. R. China; ^8^ Department of Encephalopathy Jiulongpo District Hospital of Traditional Chinese Medicine No.160, Longquan Village Chongqing City 400080 P. R. China; ^9^ Institute of Life and Health Huzhou College No.1, Bachelor Road Huzhou Zhejiang 313000 P. R. China; ^10^ Pharmacy department The Affiliated Rehabilitation Hospital of Zhejiang Chinese Medical University 2828, Binsheng Road Hangzhou Zhejiang 310000 P. R. China; ^11^ Department of Neurology The First Affiliated Hospital of Zhejiang Chinese Medical University No.54, Post and Telecommunications Road Hangzhou Zhejiang 310000 P. R. China; ^12^ Department of Cardiac‐Cerebral Diseases Yinchuan Cardiac‐Cerebral Treatment Internet Hospital No. 6 Ning'an East Lane Yinchuan Ningxia 750011 P. R. China; ^13^ Buchang Pharmaceutical Co., Ltd. No. 16 Buchang Road Xi'an Shaanxi 712046 P. R. China; ^14^ Department of Brain and Heart CO Treatment Xi'an Buchang Traditional Chinese Medicine Cardiac‐Cerebral Diseases Hospital No. 70, North Section of Labor Road Xi'an Shaanxi 712046 P. R. China; ^15^ Academy of Chinese Medical Sciences Henan University of Chinese Medicine No. 156 Jinshui East Road Zhengzhou Henan 450046 P. R. China

**Keywords:** ischemic stroke, migrasomes, proteomics, untargeted lipidomics, untargeted metabolomics

## Abstract

Ischemic stroke (IS) initiates complex systemic responses that extend beyond focal brain injury. To capture these multifaceted changes, proteomics, untargeted metabolomics, and lipidomics are integrated from IS patients spanning a range of clinical severities. This multi‐omics approach reveals distinct molecular subtypes characterized by immune activation, oxidative stress, and metabolic dysregulation. Notably, elevated levels of migrasomes are identified in patient plasma and mouse brain tissue. Proteomic profiling of these migrasomes shows enrichment in complement, coagulation, and cholesterol‐associated pathways. Functional assays further demonstrate that migrasomes derived from peripheral immune cells exacerbate ischemic injury and intensify post‐stroke inflammation. Together, these findings position migrasomes as critical drivers of IS pathophysiology and highlight them as promising targets for therapeutic intervention.

## Graphical abstract

Multi‐omics profiling of stroke patients exposes systemic molecular fingerprints of immune activation, oxidative stress and metabolic collapse. Complement and coagulation cascade, and cholesterol metabolism emerge as shared functional hallmarks of plasma migrasomes in stroke patients and migrasomes isolated from experimental stroke mice. Functional evidence shows these immune‐cell‐derived migrasomes amplify ischemic injury and inflammation, unveiling an unexpected therapeutic node.

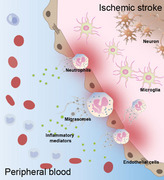

## 1 Introduction

Ischemic stroke (IS) is a prevalent cardiovascular condition with high rates of disability and mortality, imposing significant economic and social burdens on patients' healthcare systems.^[^ ^1^, ^2^ ^]^ Stroke is generally classified into ischemic and hemorrhagic types, with IS accounting for ≈62.3% of global stroke cases.^[^ ^3^ ^]^ In China, this proportion is even higher, reaching 82.6%.^[^ ^4^ ^]^ Despite its prevalence, the pathophysiological mechanisms underlying IS remain incompletely understood. Therefore, a comprehensive elucidation of the molecular characteristics and progression of IS is essential for identifying reliable blood biomarkers and advancing precision medicine approaches.

Proteomics and metabolomics—systematic analyses of proteins and metabolites in biological systems—have been widely applied in IS research. These approaches facilitate the identification of plasma biomarkers for diagnosis, prognosis, treatment evaluation, and mechanistic studies. Recently, multi‐omics strategies have offered deeper insights into the molecular mechanisms of stroke and helped uncover novel biomarkers.^[^ ^5^, ^6^, ^7^, ^8^ ^]^ Nevertheless, there is still limited knowledge regarding the molecular features and pathophysiological changes detectable through peripheral blood analyses. Identifying consistent and clinically relevant peripheral blood biomarkers for stroke remains a major challenge.

Previous studies have reported proteomic alterations related to thrombosis in post‐stroke severity^[^ ^9^ ^]^ and inflammation in post‐stroke prognosis.^[^ ^10^ ^]^ Stroke is also known to induce systemic complications—such as myocardial injury, pneumonia, splenic atrophy, and acute kidney injury—that contribute to multi‐organ damage.^[^ ^11^, ^12^ ^]^ Additionally, stroke can disrupt hepatic energy metabolism.^[^ ^13^ ^]^ Thus, investigating disease progression mechanisms, characterizing systemic, and multiorgan alterations, and exploring their underlying drivers are of critical importance. However, the precise mechanisms by which stroke severity diverges among patients are still unclear. A thorough analysis of the cellular and molecular dynamics involved in stroke development is essential to delineate disease‐related signatures, peripheral biosignatures, and potential therapeutic targets.

In this study, we aimed to enhance understanding of the molecular mechanisms underpinning the onset and progression of IS. Using data‐independent acquisition (DIA) and liquid chromatography‐mass spectrometry (LC‐MS), we quantitatively profiled proteins, small molecules, and lipid metabolites in plasma samples from patients with mild, mild‐to‐moderate, and moderate IS, with healthy individuals serving as controls. Our findings revealed distinct molecular signatures associated with IS severity. We also conducted an integrative analysis of key molecules related to the neural, immune, and endocrine systems, along with predictions of tissue damage. Furthermore, we identified and confirmed the expression of the migrasome marker protein Tspan4 across multiple organs in cerebral ischemia‐reperfusion injury (CIRI) mice. Subsequent proteomic profiling of migrasomes purified from human plasma and mouse brain underscored the pivotal contribution of immune responses after ischemic stroke. Finally, compared with CIRI mice, *Tspan9^−/−^* mice subjected to CIRI exhibited markedly attenuated inflammation, an effect reversed by exogenous migrasomes, thereby implicating migrasomes as critical mediators of early ischemic injury in stroke.

## 2 Results

### 2.1 Comprehensive Multi‐Omics Characterization in IS

**Figure 1:**
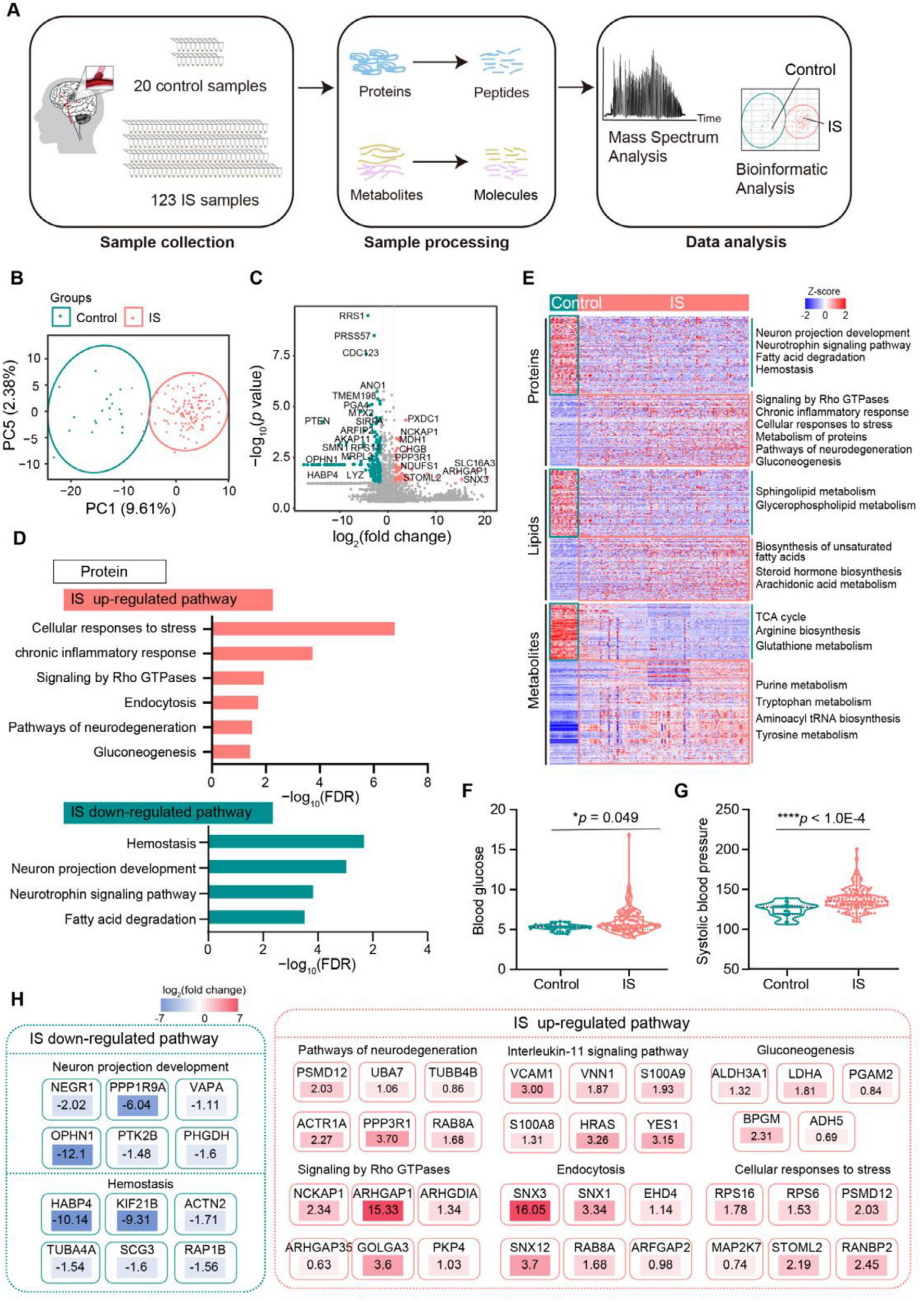
Multi‐omics profiling of IS. A) Schematic overview of the proteomics and metabolomics workflow applied in IS. B) Principal component analysis (PCA) at the protein level reveals a clear separation between IS and the control group samples. C) Volcano plot highlighting differentially expressed proteins between IS and control groups, identified using the Wilcoxon signed‐rank test. Proteins upregulated in IS are shown in red; those upregulated in controls are shown in green. D) Dominant signaling pathways at the protein level in the IS group (top panel, red) and control group (bottom panel, green). E) Heatmap illustrating key signaling pathways associated with upregulated and downregulated molecules (proteins, small molecule metabolites, and lipids) in the IS group, based on Wilcoxon signed‐rank test results. F) Violin and box plots showing significantly elevated blood glucose levels in the IS group. G) Violin and box plots showing elevated systolic blood pressure in the IS group. H) Geneboxes summarizing the major signaling pathways and representative molecules enriched in the control and IS groups. Abbreviations: IS, Ischemic stroke.

In this study, blood samples were collected from 20 healthy participants and 123 patients with IS from nine hospitals (**Figure** **1**A). The clinical characteristics of the participants are summarized in **Table** **1** and Table S1 (Supporting Information). Notably, the IS group exhibited elevated levels of red blood cell (RBC), fibrinogen (FIB), and platelets (PLTs) (Figure S1A, Supporting Information). Label‐free quantification of 143 plasma samples identified a total of 7725 protein groups with a 1% false discovery rate (FDR) at both the protein and peptide levels. Specifically, 5591 proteins were detected in the control group and 7504 in the IS group (Figure S1B, Supporting Information). The number of identified proteins was significantly higher in the IS group compared to controls (Figure S1C,D, Supporting Information). At the metabolite level, 3747 lipids were commonly detected in both groups, with an additional 20 lipids uniquely present in the IS group (Figure S1E, Supporting Information). Moreover, lipidomic profiling revealed significant differences in the number of plasma lipids between the two groups (Figure S1F, Supporting Information).

**Table 1:** Baseline characteristics of the study participants.

	Control (N=20)	IS (N=123)	*p* value
Age	47.5 ± 5.02	64.40 ± 8.54	**<0.001**
Gender (%)			0.172
Male	10 (0.5)	81 (0.66)	
Female	10 (0.5)	42 (0.34)	
Heart rate	77.30 ± 8.86	74.74 ± 9.83	0.277
Systolic blood pressure	127.50 (12.00)	135.00 (14.00)	**<0.001**
Diastolic blood pressure	86.50 (9.75)	80.00 (11.00)	0.098
RBC	4.82 (0.40)	4.48 (0.66)	**0.001**
WBC	5.81 (1.42)	6.08 (1.93)	0.441
HB	143.50 (30.50)	139.00 (18.00)	0.875
PLT	268.80±77.49	220.90±65.15	**0.004**
ALT	22.00 (13.75)	24.00 (17.00)	**0.012**
AST	21.50 (8.50)	24.00 (10.90)	0.051
ALP	65.5 (23.00)	80.50 (32.50)	**0.001**
TBIL	10.00 (3.65)	11.33 (5.88)	0.121
GGT	19.50 (11.00)	29.00 (26.00)	**<0.001**
BUN	4.72 (1.43)	4.91 (2.16)	0.998
Cr	63.50 (26.50)	67.00 (29.80)	0.364
PT	13 (1.07)	11.4 (1.44)	**<0.001**
APTT	34.50 (4.20)	26.80 (5.40)	**<0.001**
TT	15.85 (0.77)	16.6 (1.93)	**0.014**
FIB	3.03 (0.40)	3.08 (1.59)	0.769
GLU	5.34 (0.42)	5.56 (1.74)	**0.049**

**Abbreviatures**. ALP, Alkaline phosphatase; ALT, Alanine aminotransferase; APTT, Activated partial thromboplastin time; AST, Aspartate transaminase; BUN, Blood urea nitrogen; Cr, Creatinine; FIB, Fibrinogen; GGT, Gamma‐Glutamyl Transferase; GLU, Glucose; HB, Haemoglobin; PLT, Platelet; PT, Prothrombin time; RBC, Red blood cell; TBIL, Total bilirubin; TT, Thrombin time; WBC, White blood cell.

Principal component analysis (PCA) based on proteomic and metabolomic data demonstrated a clear separation between IS and control samples at the protein, lipid, and small‐molecule levels (Figure 1B; Figure S1G, Supporting Information). Pathway enrichment analysis revealed the upregulation of signaling pathways associated with Rho GTPases, chronic inflammatory responses, and the biosynthesis of reactive oxygen species and unsaturated fatty acids. Conversely, pathways such as glutathione metabolism were downregulated, indicating increased cell migration, heightened inflammation, and oxidative stress following stroke. Additionally, the upregulation of gluconeogenesis and the downregulation of pathways related to fatty acid degradation, sphingolipid metabolism, arginine metabolism, and tyrosine metabolism suggest widespread disruptions in glycolipid and amino acid metabolism. These pathways are closely linked to the tricarboxylic acid (TCA) cycle, which was also found to be downregulated. Collectively, these abnormalities point toward mitochondrial dysfunction and subsequent impairment in energy metabolism^[^ ^14^ ^]^ (Figure 1D,E).

Further clinical analyses revealed significantly higher blood glucose levels (*P* = 0.049) and systolic blood pressure (*P*<1.0E‐4) in the IS group compared to the control group (Figure 1F,G). In addition, cell proliferation‐related metabolites such as xanthine and deoxyguanosine were markedly upregulated in the IS group (Figure S1J, Supporting Information), suggesting a potential involvement of cell cycle regulation in stroke pathology.

### 2.2 Proteomic and Metabolomic Clusters in the IS Group

**Figure 2:**
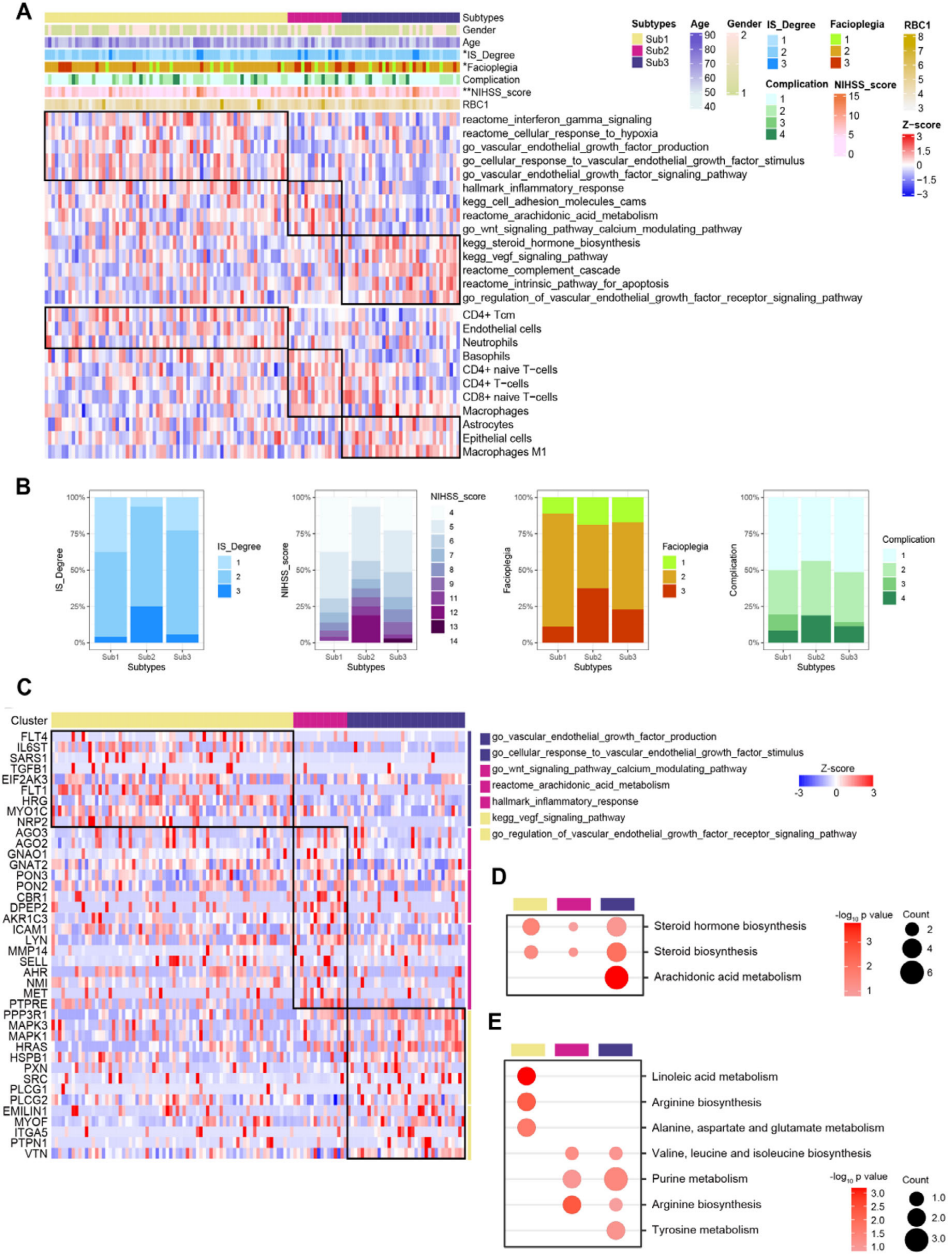
Molecular characteristics of patients with IS. A) Heatmap illustrating the association between proteomic subtypes, pathological features, pathways, and immune cell types identified by consensus clustering. Statistical significance was assessed using Fisher's exact tests. B) Genebox summarizing the key pathological signals characteristic of each proteomic subtype. C) Heatmap of differentially expressed proteins across samples grouped into three clusters based on proteomic results. D) Bubble plot representing the major lipid metabolic pathways enriched in the three sample clusters defined by lipid metabolomics. E) Bubble plot depicting the predominant small‐molecule metabolic pathways associated with the three clusters identified through small‐molecule metabolomics. Abbreviations: IS, Ischemic stroke.

To evaluate molecular heterogeneity among patients with IS, consensus clustering was performed based on proteomic data from all IS samples. The analysis revealed a significant correlation between molecular subtypes and stroke severity, as measured by the National Institutes of Health Stroke Scale (NIHSS). Notably, patients classified under proteomic subtype 2 exhibited a higher proportion of elevated NIHSS scores and facial palsy (facioplegia, **Figure** **2**A,B). Differential expression analysis demonstrated that proteomic subtype 2 was characterized by upregulation of proteins involved in inflammatory responses, cell adhesion molecules (CAMs), arachidonic acid metabolism, and calcium‐related components of the Wnt signaling pathway. Immune cell infiltration profiling further revealed that patients with higher NIHSS scores had increased levels of macrophages, basophils, CD4+ T cells, and naïve CD4+/CD8+ T cells compared to other IS patients (Figure 2A,B). Key pathway targets associated with this subtype are presented in Figure 2C. At the metabolomic level, the data pointed to the involvement of steroid hormone biosynthesis in the disease process. Additional metabolic pathways enriched in subtype 2 included arginine biosynthesis, purine metabolism, and branched‐chain amino acid biosynthesis (valine, leucine, and isoleucine) (Figure 2D,E). These findings suggest that this molecular subtype is characterized by heightened inflammation, immune activation, and distinct metabolic dysregulation, which may contribute to increased stroke severity.

### 2.3 Diverse Characterization Across IS Severity Levels

**Figure 3:**
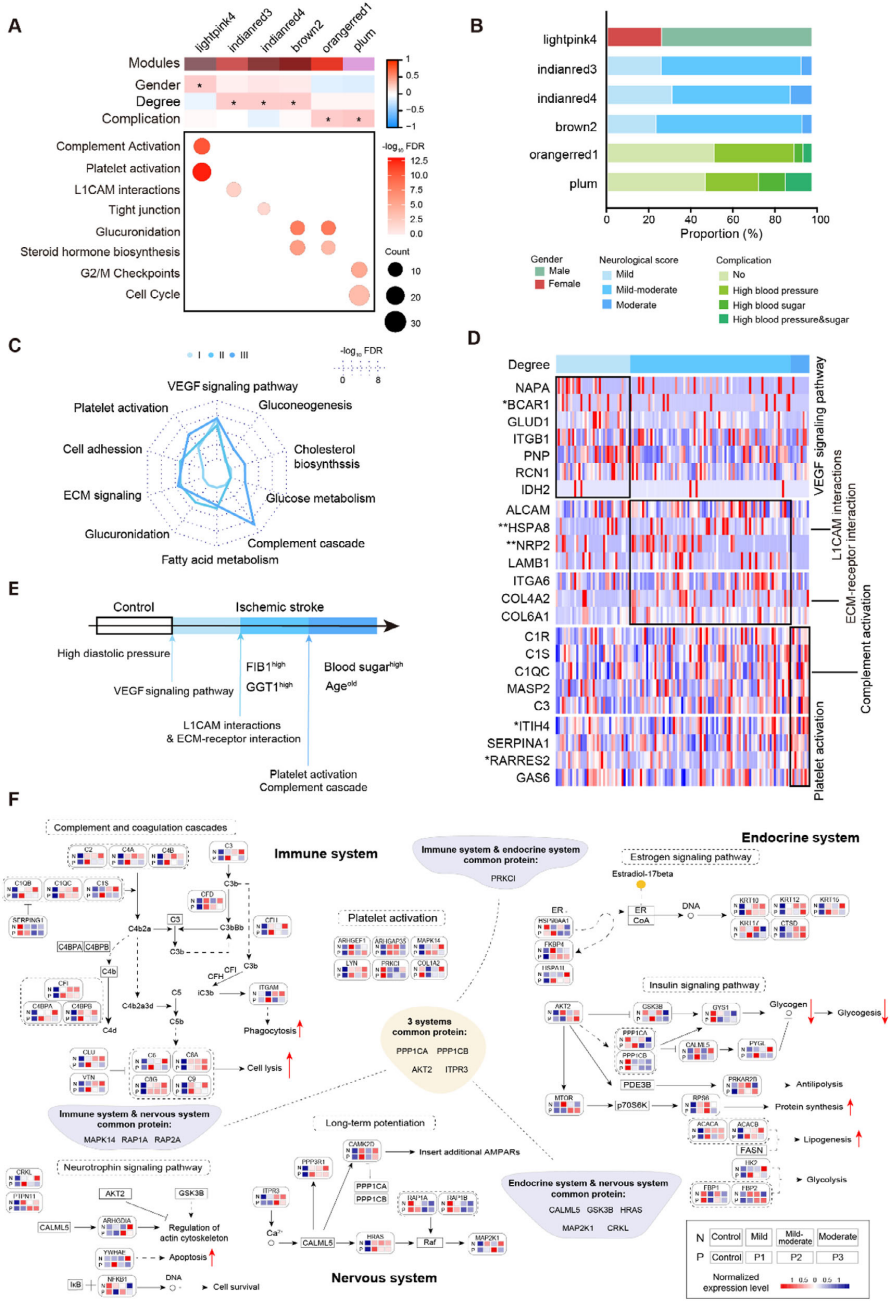
Protein differences associated with the pathophysiology of IS. A) Top: Heatmap illustrating the correlations between coexpression modules and pathological features identified by weighted gene coexpression network analysis (WGCNA). Bottom: Bubble plot showing the primary signaling pathways enriched within each module based on associated proteins. B) Bar chart representing the distribution of different pathological subpopulations within modules significantly correlated with IS traits. C) Summary of key molecular signals observed across IS patients stratified by neurological scores. D) Heatmap depicting expression patterns of proteins and associated signaling pathways in patients with mild‐to‐moderate IS, analyzed via the Kruskal–Wallis test. E) Summary illustrating distinct clinical and molecular characteristics among stroke patients categorized by National Institutes of Health Stroke Scale (NIHSS) scores. F) Network diagrams of the immune, endocrine, and nervous system pathway. N represents average protein expression levels in peripheral blood from healthy controls and IS patients stratified by disease severity (mild, mild–moderate, moderate). P denotes average protein expression levels in peripheral blood at different disease time points (7, 8–14, and 15–28 days post‐stroke). Abbreviations: IS, Ischemic stroke.

We applied weighted gene coexpression network analysis (WGCNA) at both the proteomic and metabolic levels to identify molecular modules associated with the physiological and pathological features of IS. Five modules demonstrated significant correlations with key pathological traits of IS (**Figure** **3**A,B). Among these, the lightpink4 module was predominantly linked to sex, with a higher proportion of male stroke patients. This module´s molecules were mainly involved in complement activation and platelet signaling pathways. The orangerred1 and plum modules correlated primarily with complications such as hypertension and hyperglycemia in IS patients. Molecules in these modules were enriched in glycosylation processes, steroid hormone biosynthesis, and cell cycle regulation. The indianred3, indianred4, and brown2 modules showed strong associations with IS severity; their key pathways included L1CAM interactions, tight junctions, glycosylation, and steroid hormone biosynthesis (Figure 3A). Our analysis suggested that vascular damage is widespread among IS patients, with the vascular endothelial growth factor (VEGF) signaling pathway enriched in mild IS cases and persisting through to severe stages. In moderate IS, major pathways involved platelet activation, complement cascade, cell adhesion, extracellular matrix (ECM) signaling, glucuronidation, and cholesterol biosynthesis (Figure 3C,D), indicating that disruptions in glycolipid metabolism and immune dysfunction play critical roles in disease severity. At the metabolic level, steroid biosynthesis and arachidonic acid metabolism were identified as key pathways, highlighting the interplay between lipids and endocrine hormones across different stroke severities (Figure S2C,D, Supporting Information).

Integration with clinical data revealed that the moderate IS group was characterized by older age, shorter time since stroke onset, higher blood glucose levels, and lower diastolic blood pressure (Figure 3E; Figure S2A,B, Supporting Information). Further investigation into DEPs stratified by IS severity and onset time employed the Kyoto Encyclopedia of Genes and Genomes (KEGG) database for pathway classification. Enriched pathways among both upregulated and downregulated DEPs overlapped considerably (Figure S2E, Supporting Information) and predominantly involved three major biological systems (Figure S2F, Supporting Information). Expression levels of proteins in these pathways were significantly elevated in patients with the highest disease severity and earliest onset. Functionally, these pathways suggest that activation of the complement system may enhance phagocytosis and induce cell lysis. Meanwhile, activation of the insulin signaling pathway likely influences lipid homeostasis by disrupting glycolysis and inhibiting glycogen synthesis. Activation of neurotrophic factor signaling pathways may modulate cell survival and apoptosis (Figure 3F). In summary, our results highlight extracellular‐matrix (ECM) signaling, cell adhesion, and the complement and coagulation cascade as key drivers of IS progression. Notably, these pathways are all related to migrasomes.^[^ ^15^, ^16^, ^17^ ^]^ This study will focus on migrasomes as a starting point to explore their underlying mechanisms in stroke development.

### 2.4 Tissue Damage Tracking After IS

**Figure 4:**
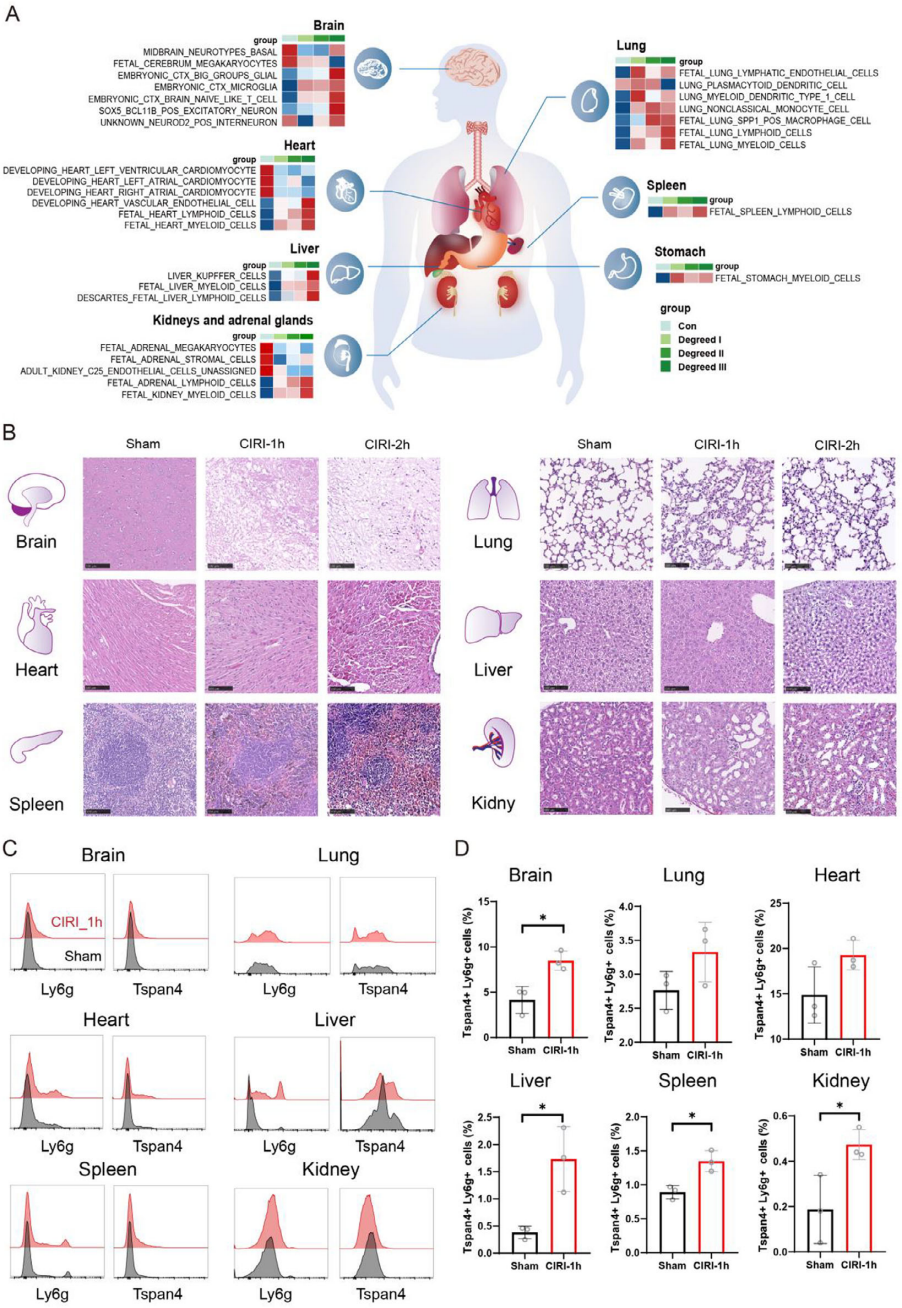
Changes in tissue‐related proteins in patients with IS. A) Heatmaps displaying mean intragroup gene set variation analysis (GSVA) enrichment scores for tissue‐specific proteins related to the brain, heart, lung, spleen, adrenal gland, and kidney. B) Representative hematoxylin and eosin (H&E) stained histopathological images of the brain, heart, lung, liver, spleen, and kidney from mice subjected to CIRI for 1 h (CIRI‐1 h) and 2 h (CIRI‐2 h), alongside age‐ and sex‐matched controls. C) Flow cytometric analysis showing positive expression of Ly6g and Tspan4 in the brain, heart, liver, spleen, lung, and kidney. D) Quantification of Ly6g^+^, Tspan4^+^, and double‐positive Ly6g^+^Tspan4^+^ cell populations by flow cytometry. Three animals per group were analyzed. Data are presented as mean ± standard deviation (SD), with statistical significance assessed (Student's unpaired *t* test). ^*^ *p* < 0.05, ^**^ *p*< 0.01, ^***^ *p*< 0.001, ^****^ *p*< 0.0001. Fluorescent channels used: Tspan4 (PE), Ly6g (PerCP). Abbreviations: CIRI, cerebral ischemia‐reperfusion injury.

Acute IS can cause autonomic dysfunction, activation of the hypothalamic‐pituitary‐adrenal (HPA) axis, systemic immune imbalance, and widespread extracerebral pathophysiological changes. Collectively, these effects can induce or exacerbate structural damage and functional impairments across multiple peripheral organs.^[^ ^12^ ^]^ Changes in the expression of tissue‐specific proteins detected in the peripheral blood of patients with varying IS severity suggest multi‐organ involvement, including the brain, heart, lung, liver, spleen, adrenal gland, and kidneys (**Figure** **4**A).

To further explore these systemic effects, a mouse CIRI model was established with different severities (1 h and 2 h ischemia followed by 1 day of reperfusion). Histopathological analysis revealed that neuronal cells in the cerebral cortex of CIRI mice exhibited swelling, disrupted arrangement, nuclear condensation, severe vacuolization, and a marked reduction in neuronal density compared to controls. Liver tissues show more evident damage, including disorganized nuclei, hepatocyte edema, ballooning degeneration, cytoplasmic loosening with abundant intracellular particles, and frequent nuclear condensation and lysis. In the lungs, CIRI mice exhibited more severe pulmonary hemorrhage, characterized by extravasation of erythrocytes and plasma proteins into alveolar spaces, reduced alveolar volume, thickened interstitium, and dilated capillaries in alveolar walls, indicating compromised ventilation. Renal tubules appeared dilated with enlarged lumens, vacuolization of tubular epithelial cells, luminal cell detachment, and interstitial inflammatory infiltration. The spleen showed pronounced bruising, with extensive erythrocyte extravasation between splenic trabeculae of the red pulp and an increased presence of ferritin‐containing macrophage granules (brownish‐black/yellowish), indicating massive erythrocyte destruction (Figure 4B).

Recent studies have identified migrasomes—organelles released by migrasomes that facilitate intercellular communication^[^ ^18^, ^19^, ^20^ ^]^—as key players in various diseases.^[^ ^17^, ^21^ ^]^ However, their role in stroke pathology remains poorly characterized. Tspan4 has been recognized as the primary marker of migrasomes in this context.^[^ ^22^ ^]^ Accordingly, we investigated the association between migrasomes and multiple organs in stroke. Flow‐cytometric revealed a significant, progressive increase in Tspan4⁺ Ly6g⁺ cell populations in the brain, liver, spleen, and kidney correlating with the duration of cerebral ischemia in the mouse stroke model (*p*<0.05) (Figure 4C,D). Immunohistochemical analysis demonstrated co‐localization of Tspan4⁺ signals with CD31+ endothelial markers across multiple organs. Compared to controls, Tspan4 expression was downregulated in the lungs and liver but upregulated in the spleen and kidney (Figure S3A–F, Supporting Information).

### 2.5 Increased Migrasomes in CIRI Mouse Models

**Figure 5:**
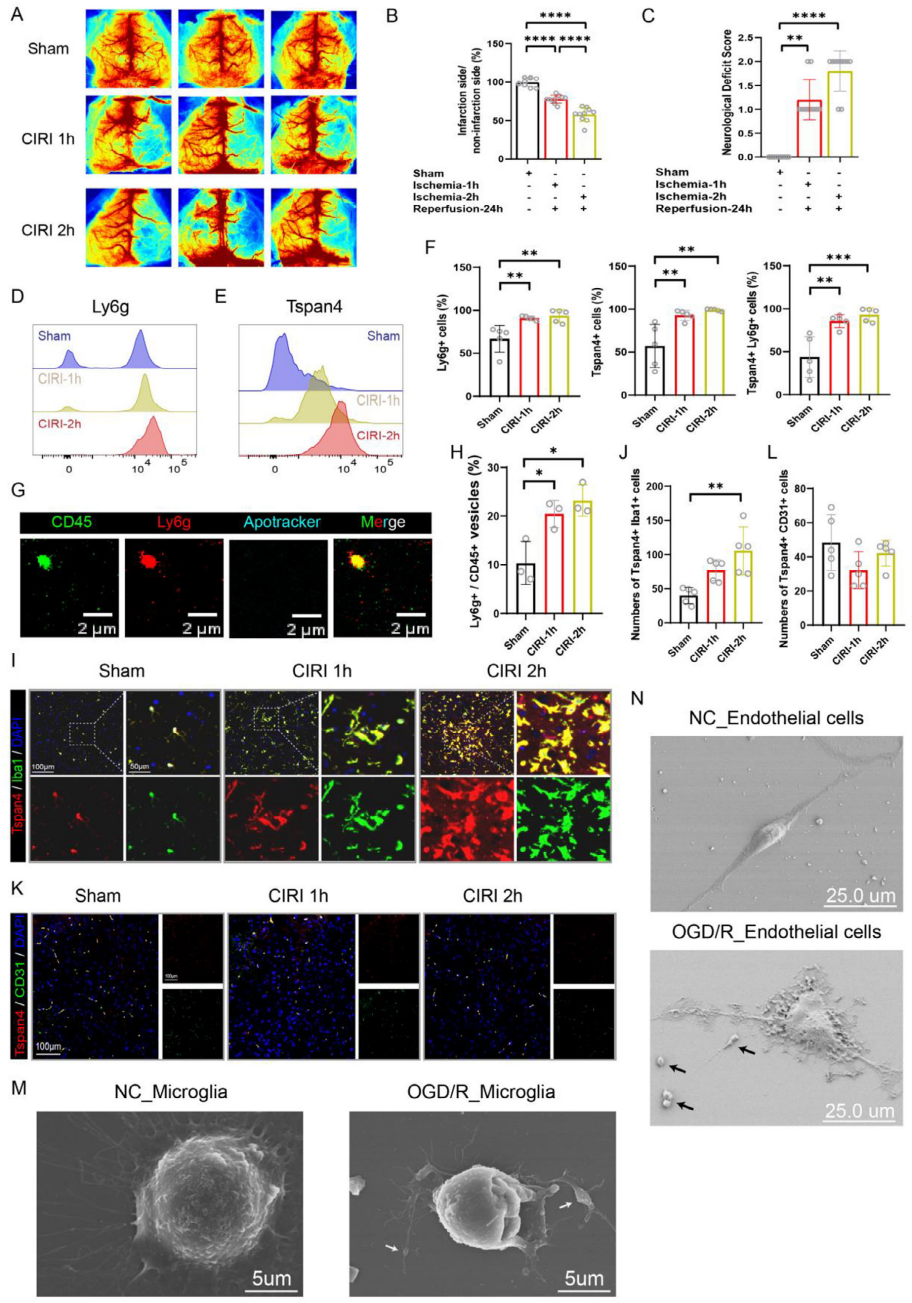
Multihistopathological findings and altered circulating immunological features of CIRI mice. A) Representative images from laser speckle contrast imaging (LSCI). B) Quantification of perfusion volumes on the ischemic vs non‐ischemic sides using LSCI (*n* = 10 per group). C) Neurological Deficit Score for each group (*n* = 10 per group). D) Flow cytometry analysis of Ly6g expression in peripheral blood cells. E) Flow cytometry analysis of Tspan4 expression in peripheral blood cells. F) Quantification of Ly6g^+^, Tspan4^+^, and double‐positive Ly6g^+^Tspan4^+^ cells by flow cytometry. Samples from five animals per group. Fluorescent channels used: Tspan4 (PE), Ly6g (PerCP). G) Dragonfly microscope images showing migrasomes in peripheral blood. Scale bar: 2 µm. The non‐exposure of phosphatidylserine on the migrasomal surface was confirmed by the absence of Apotracker staining, thereby excluding contamination by non‐migrasomal microvesicles, exosomes, or cellular debris of other origins. Fluorescent channels used: CD45 (green), Ly6g (red), Apotracker (blue). H) Quantification of plasma CD45^+^ and Ly6g^+^ migrasomes (n=3 per group). I,J) Left panels: Representative multiplex immunohistochemistry images of Tspan4 (red), Iba1 (green), and DAPI (blue) in brain tissue. Right panels: Quantification of Tspan4^+^ Iba1^+^ cells. Scale bar: 100 µm (*n* = 5 per group). K,L) Left panels: Representative multiplex immunohistochemistry images of Tspan4 (red), CD31 (green), and DAPI (blue) in brain tissue. Right panels: Quantification of Tspan4^+^ CD31^+^ cells. Scale bar: 100 µm (*n* = 5 per group). M) Representative scanning electron microscopy (SEM) images of HMC3 cells subjected to 3 h oxygen‐glucose deprivation followed by 21 h reperfusion compared with untreated controls. Scale bar: 5 µm. N) Representative SEM images of bEnd.3 cells treated similarly to HMC3 cells. Scale bar: 25 µm. All data are expressed as means ± standard deviation (SD). Statistical significance: ^*^ *p* < 0.05, ^**^ *p* < 0.01, ^***^ *p* < 0.001, ^****^ *p *< 0.0001 by one‐way ANOVA followed by Tukey's multiple comparison tests. Abbreviation: CIRI, cerebral ischemia‐reperfusion injury.

As described previously, we stablished mouse CIRI models with varying ischemic durations. Validation of the model using laser Doppler blood flow imaging and neurological scoring confirmed its effectiveness (**Figure** **5**A–C). Previous work by the Jiao group identified neutrophils as the primary source of migrasomes in peripheral blood.^[^ ^23^ ^]^ Consistent with this, flow cytometry revealed a significant increase in Tspan4+ Ly6g+ cells in mouse peripheral blood, which correlated positively with ischemia duration (*p*< 0.01) (Figure 5D–F). Further analysis quantified total immune cells and neutrophil‐derived migrasomes in peripheral blood. The ratio of CD45^+^ to Ly6g^+^ cells rose as ischemic time increased (Figure 5G,H).

To identify the predominant migrasomes in the brain post‐stroke, we performed colocalization staining. Tspan4^+^ signals co‐localized with both Iba1^+^ microglia/macrophages and CD31^+^ endothelial cells. Notably, after 1 day of reperfusion, the number of Tspan4^+^Iba1^+^ cells significantly increased with ischemia duration (*p*< 0.01) (Figure 5I,J). Conversely, Tspan4^+^CD31^+^ cells numbers decreased (Figure 5K,L). Scanning electron microscopy further confirmed the presence of elliptical vesicles consistent with migrasomes in retraction fibers of HMC3 microglial and bEnd.3 endothelial cells subjected to oxygen‐glucose deprivation/reperfusion (OGD/R) injury (Figure 5M,N).

### 2.6 Migrasomal Proteome Signature in CIRI Mouse Brains

To further investigate the functional role of migrasomes following stroke, we isolated migrasomes from the mouse brain and performed proteomic validation (Figure S4A, Supporting Information). Migrasome identity was first confirmed using transmission electron microscopy (TEM) (Figure S4B, Supporting Information). Proteomic analysis revealed substantial differences in protein expression between the sham, CIRI‐1 h, and CIRI‐2 h groups (Figure S4C, Supporting Information). Using a threshold of log2FC≥1 and *p*<0.05, the CIRI‐1 h group exhibited significant upregulation of 203 mitochondrial proteins, 101 nuclear proteins, and 50 ECM proteins compared with the sham group. Similarly, the CIRI‐2 h group showed upregulation of 25 mitochondrial proteins, 19 nuclear proteins, and 27 ECM proteins relative to sham (Figure S5, Supporting Information). Pathway enrichment analysis of the differentially expressed proteins across the three groups indicated that the upregulated proteins were mainly associated with the complement and coagulation cascades, as well as cholesterol metabolism. In contrast, the downregulated proteins were predominantly involved in neural developmental pathways (Figure S4D–I, Supporting Information).

### 2.7 Migrasomal Proteome Characterization of Plasma and Brain Tissue

**Figure 6:**
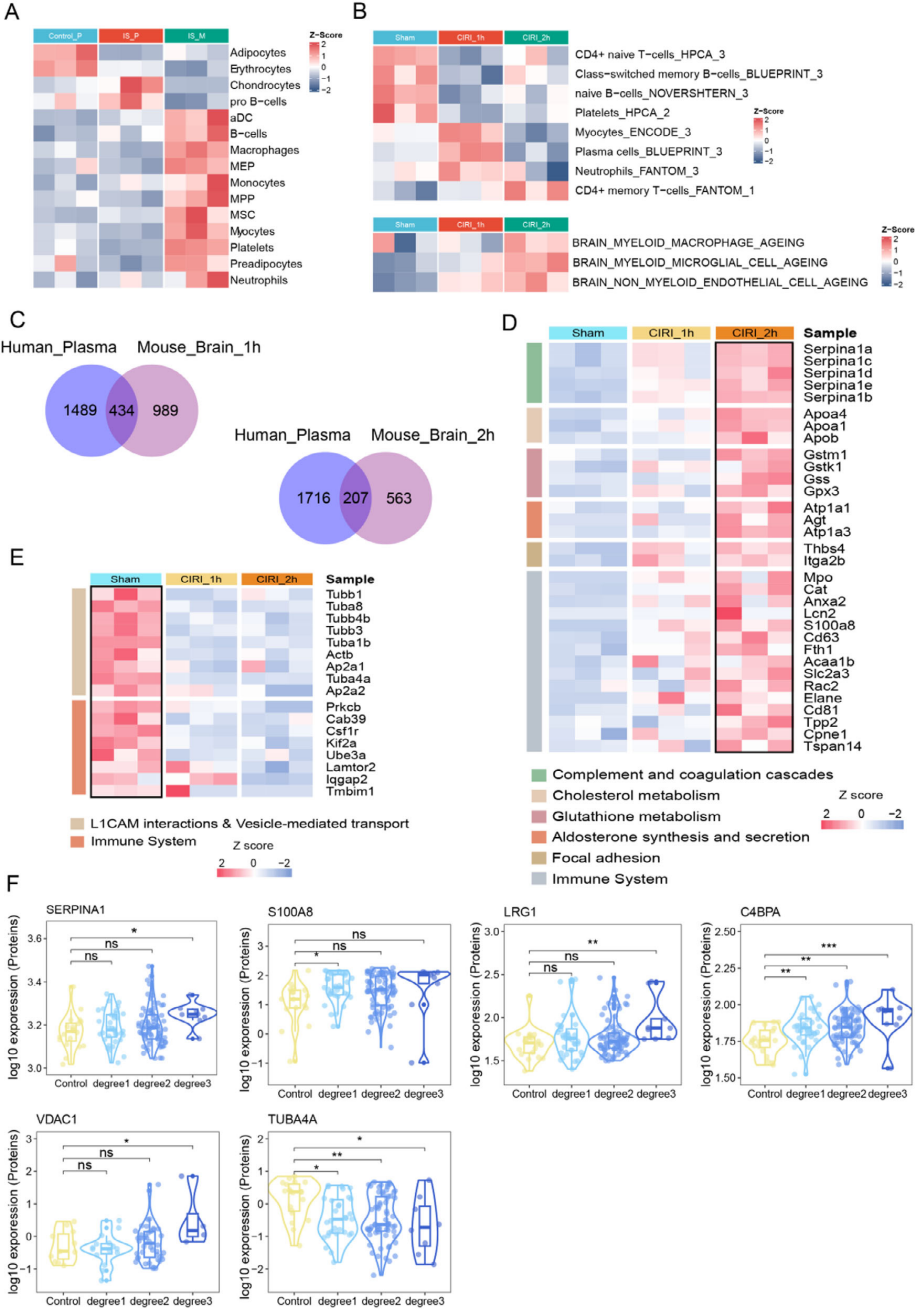
Functional proteomic analysis of peripheral blood‐ and brain‐derived migrasomes in patients and CIRI mouse models. A) Heatmap illustrating cell type enrichment across the proteomes of age‐ and sex‐matched healthy controls, IS patient plasma, and migrasomes isolated from IS patient plasma. B) Heatmap showing cell type enrichment in brain‐derived migrasomes across sham, CIRI‐1h, and CIRI‐2h mouse groups. C) Venn diagrams displaying the overlap of DEPs between control plasma vs. patient plasma migrasome proteomes and differentially expressed proteins (DEPs) from sham vs CIRI‐1h (left) and sham vs. CIRI‐2h (right). Statistical analysis was performed using unpaired two‐tailed t‐tests. D,E) Heatmaps summarizing the enriched signaling pathways associated with proteins showing upward (D) or downward (E) expression trends across sham, CIRI‐1h, and CIRI‐2h groups. F) Violin plot demonstrating the correlation between NIH Stroke Score (NIHSS) scores and protein expression levels in patient plasma, with statistical significance assessed by Dunnett's t test. Abbreviation: IS, Ischemic stroke. CIRI, cerebral ischemia‐reperfusion model.

To further investigate the potential functions of migrasomes and identify their cellular origins, we analyzed the proteomic profiles of peripheral blood‐derived migrasomes from three ischemic stroke patients. Comparative analysis was performed among three groups: the plasma proteome of age‐ and sex‐matched healthy controls, the plasma proteome of IS patients, and the proteome of isolated migrasomes from patient plasma. The results revealed that multiple cell types—particularly neutrophils and monocytes—were the predominant sources of circulating migrasomes in patients (**Figure** **6**A). In the mouse brain, the main contributors to migrasomal origin were microglia, endothelial cells, and neutrophils (Figure 6B).

Next, we performed differential protein analysis between the plasma proteome of controls and the migrasomal proteome from patient plasma. These differentially expressed proteins were compared with dose identified in the sham vs CIRI‐1 h group, and in the sham vs CIRI‐2 h group, yielding 434 and 207 shared differential proteins, respectively (Figure 6C). Proteins exhibiting upregulated trends were mainly involved in complement and coagulation cascades, cholesterol metabolism, and immune system regulation (Figure 6D). In contrast, downregulated proteins were primarily enriched in L1CAM‐mediated interactions, vesicle‐mediated transport, and immune‐related pathways (Figure 6E). Moreover, we identified proteins whose expression levels correlated with disease severity. Among the most prominent were inflammatory and coagulation‐related proteins such as Serpin Family A Member 1 (SERPINA1),^[^ ^23^, ^24^ ^]^ inflammatory mediators S100 Calcium Binding Protein A8 (S100A8),^[^ ^25^ ^]^ angiogenesis‐associated Leucine‐Rich Alpha‐2‐Glycoprotein 1 (LRG1),^[^ ^26^, ^27^ ^]^ immune‐related Complement Component 4 Binding Protein Alpha (C4BPA), mitochondrial membrane protein Voltage‐Dependent Anion Channel 1 (VDAC1),^[^ ^28^ ^]^ and cytoskeletal protein Tubulin Alpha 4A (TUBA4A) (Figure 6F).

### 2.8 Migrasomes Derived from Peripheral Immune Cells Exacerbate Early Inflammation in CIRI

**Figure 7:**
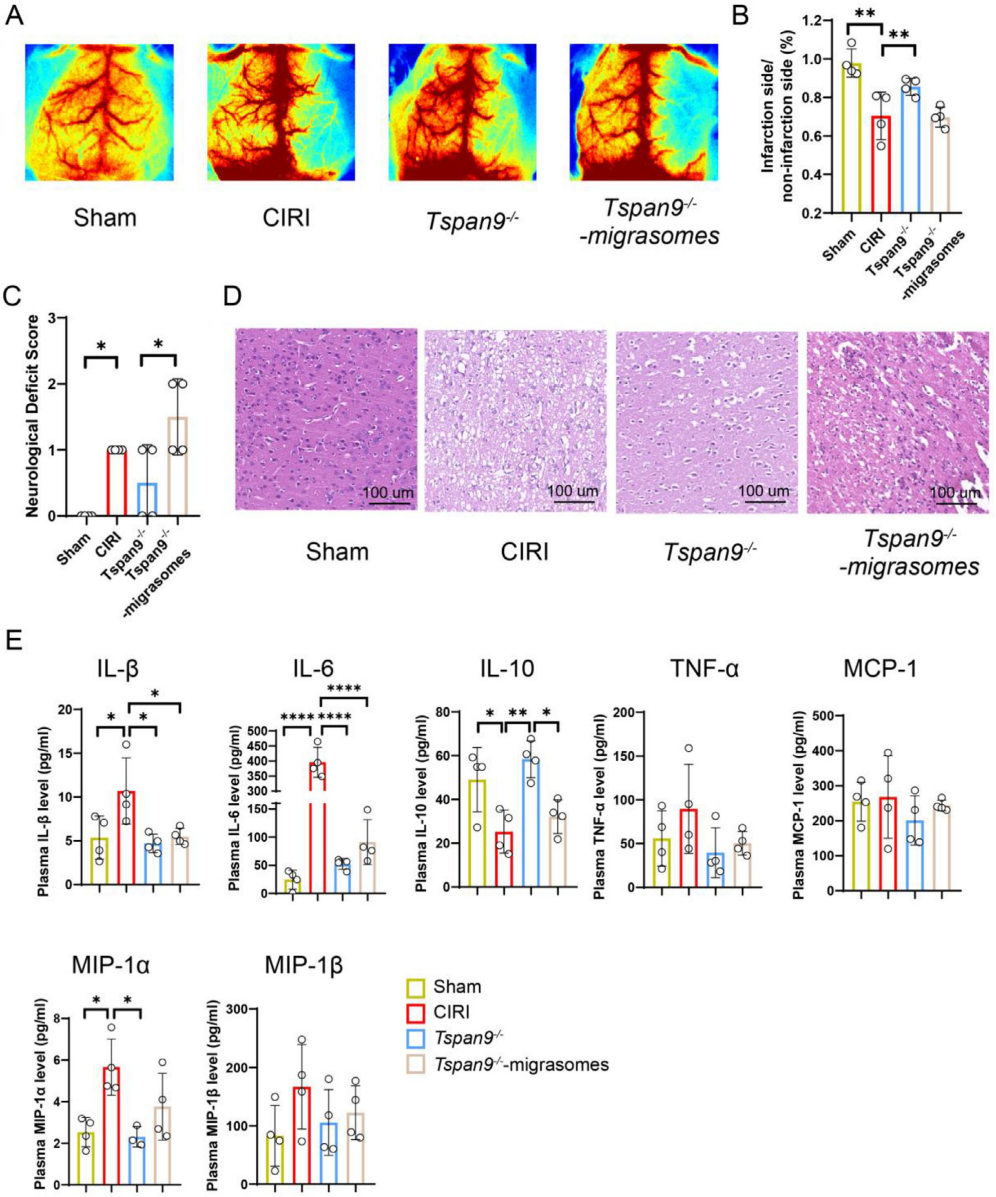
Migrasomes promote early inflammation in the CIRI model. A) Representative images from laser speckle contrast imaging. 1 h after establishing the CIRI model, mice in the *Tspan9^−/^* ^−^‐migrasomes group were intravenously injected with migrasomes derived from peripheral blood immune cells (2 × 10^7^ per mouse). B) Quatification of perfusion volume on the ischemic side relative to the non‐ischemic side, based on laser speckle contrast imaging. n=4 per group. C) Neurological Deficit Score. *n* = 4 per group. D) Representative hematoxylin and eosin (H&E) stained histopathological images of the brain from mice. E) Plasma levels of IL‐1β, IL‐6, IL‐10, TNF‐α, MCP‐1, MIP‐1α, and MIP‐1β. *n* = 4 per group. All statistical data are presented as mean ± standard deviation (SD). ^*^ *p *< 0.05, ^**^ *p* < 0.01, ^***^ *p* < 0.001, ^****^ *p *< 0.0001 by one‐way ANOVA followed by Tukey's multiple comparison test. CIRI, cerebral ischemia‐reperfusion model.

Based on our analysis of the functional characteristics of migrasomes, we hypothesized that these vesicles may contribute to the release of substantial immune signals during the acute phase of stroke. To test this, we utilized *Tspan9^−/−^* mice, which exhibit impaired migrasome formation.^[^ ^23^ ^]^ Additionally, the role of peripheral immune cell‐derived migrasomes was evaluated by intravenous injection after CIRI induction. *Tspan9* knockout significantly improved cerebral blood flow perfusion following CIRI, whereas perfusion was notably reduced in mice that received *Tspan9^−/−^*‐derived migrasomes (**Figure** **7**A,B). Neurological deficits were improved in mice that received *Tspan9^−/^* ^−^‐derived migrasomes among the groups (Figure 7C). Histopathological examination showed only scant cell death in the sham group, whereas CIRI precipitated extensive cell loss. Genetic deletion of *Tspan9* mitigated cell death; however, subsequent administration of exogenous migrasomes reversed this phenomenon. These findings indicate that migrasomes contribute to the pathological progression of CIRI (Figure 7D). Next, we measured plasma cytokine levels. Compared to the CIRI group, *Tspan9^−/−^* mice exhibited significantly lower levels of pro‐inflammatory cytokines IL‐1β, IL‐6, and MIP‐1α (*p *< 0.05), and a significantly higher level of the anti‐inflammatory cytokine IL‐10 (*p*< 0.05) (Figure 7E).

## 3 Discussion

In this comprehensive multi‐omics study, we provide a system‐level characterization of IS pathogenesis by integrating proteomic and metabolomic profiling with in vivo validation in a CIRI mouse model. Our findings highlight widespread systemic dysregulation triggered by IS, involving immune activation, oxidative stress, and metabolic disturbances. Notably, our data point to a crucial role for cell adhesion and migration processes in IS pathology.

We identified neutrophil‐derived migrasomes as a key component in both patients’ peripheral blood and the brains of CIRI model mice. Experimental validation demonstrated a positive correlation between ischemic duration and the number of neutrophil‐derived migrasomes in peripheral blood. Following CIRI, Tspan4⁺ neutrophils increased in multiple organs. Moreover, genetic ablation of *Tspan9* resulted in improved cerebral perfusion in the ischemic hemisphere, ameliorated histopathological injury, and reduced peripheral inflammation. These findings suggest that extracellular vesicles derived from migrating immune cells—particularly migrasomes—play a central role in mediating early systemic inflammation after ischemic injury.

Our analyses also revealed that activation of the complement cascade, endothelial injury, and enhanced cell migration are closely associated with stroke severity. Mechanistically, VEGF signaling appears to initiate disease progression, followed by increased adhesion and extracellular matrix (ECM) signaling, ultimately leading to upregulation of cholesterol synthesis and complement cascade activation. Migrasomes, which form from retraction fiber breakage during cell adhesion and migration, are tightly linked to these processes.^[^ ^29^ ^]^ Integrins enriched at the base of migrasomes (e.g., α5β1–fibronectin interactions) mediate their anchorage and formation.^[^ ^15^ ^]^ Upon vascular injury, migrasomes rapidly accumulate at lesion sites, where surface integrins facilitate platelet binding, activation, and thrombus formation.^[^ ^16^ ^]^ Additionally, migrasomes can activate the complement system, promoting complement‐dependent cytotoxicity that disrupts the blood–brain barrier and worsens injury.^[^ ^17^ ^]^ Thus, migrasomes serve as key integrators of ECM‐derived adhesive cues and complement–coagulation signaling, contributing to both inflammatory and thrombotic processes. Cholesterol also plays a critical role in migrasome biogenesis.^[^ ^30^ ^]^ Our proteomic analysis of brain‐ and blood‐derived migrasomes revealed enrichment in proteins associated with complement activation, coagulation cascades, and cholesterol metabolism. These findings suggest that migrasomes are not only byproducts of cellular migration but also active participants in peripheral immune and thrombotic responses, potentially driving the onset and progression of IS.

Consistent with previous reports,^[^ ^31^ ^]^ our findings suggest that post‐stroke pathology involves multi‐organ damage, immune cell infiltration, and widespread systemic effects. While cerebral injury remains the primary clinical manifestation, stroke is frequently associated with complications in other organs. Clinical studies have reported common post‐stroke complications such as respiratory tract infections,^[^ ^32^ ^]^ hepatic impairment,^[^ ^33^ ^]^ renal dysfunction,^[^ ^34^, ^35^ ^]^ and splenic atrophy.^[^ ^36^ ^]^ Experimental data have also revealed cardiac fibrosis and functional decline following stroke.^[^ ^37^ ^]^

Animal models of experimental stroke have demonstrated an increased incidence of pulmonary infections,^[^ ^38^ ^]^ along with impaired phagocytic activity in lung macrophages.^[^ ^39^ ^]^ Notably, the severity of pulmonary infection is positively correlated with the extent of ischemic damage.^[^ ^40^ ^]^ Stroke has also been shown to promote hepatic injury and disturbances in lipid metabolism. Interestingly, obesity does not appear to exacerbate stroke‐induced liver injury.^[^ ^41^ ^]^ In the spleen, although macrophage density increases, their expression of immune function‐related genes is diminished following experimental stroke.^[^ ^42^ ^]^ Additionally, stroke induces renal insufficiency and heightened sympathetic nerve activity in murine models.^[^ ^43^ ^]^ In this study, we observed marked pathological changes in multiple organ systems—including the brain, lungs, liver, kidneys, and spleen—during the acute phase of ischemic stroke. Notably, expression levels of Tspan4 and Ly6g were elevated across these organs after CIRI, suggesting systemic activation of immune and migratory processes.

Moreover, we identified microglia and endothelial cells as the predominant sources of migrasomes within the brain. Previous studies provide mechanistic insights into this phenomenon. For instance, Zhang et al. reported that an oxidized low‐density lipoprotein (ox‐LDL)‐induced in vitro model of atherosclerosis prompted endothelial cells to produce migrasomes.^[^ ^44^ ^]^ These migrasomes carried amyloid precursor protein (APP), which promoted macrophage polarization toward the pro‐inflammatory M1 phenotype. Similarly, Clostridium difficile toxin B subtype 3 has been shown to stimulate migrasome formation in endothelial cells.^[^ ^45^ ^]^ Interactions between endothelial cells and microglia are known to affect blood–brain barrier (BBB) integrity. Endothelial cells can promote M1 polarization of microglia, contributing to neuronal injury.^[^ ^46^ ^]^ Conversely, mononuclear‐derived macrophages may exert protective effects. In a murine model of ischemia–reperfusion injury, macrophage‐derived IL‐33 was found to reduce endothelial cell damage and BBB leakage.^[^ ^47^ ^]^ Our in vitro OGD/R (oxygen–glucose deprivation/reoxygenation) experiments using scanning electron microscopy revealed the presence of migrasome‐like structures in both microglia and endothelial cells. These migrasomes may facilitate intercellular communication and material exchange, potentially contributing to disease progression. Together, these findings offer new insights into the role of migrasomes in post‐stroke organ crosstalk and provide a novel framework for future studies exploring intercellular signaling after ischemic injury.

Several core proteins enriched in migrasomes—including SERPINA1, S100A8, LRG1, C4BPA, VDAC1, and TUBA4A—were strongly associated with stroke severity, underscoring their potential as biomarkers or therapeutic targets. For example, SERPINA1 has been identified as a biomarker of high‐density lipoprotein cholesterol associated with platelet activation during the acute phase of stroke.^[^ ^5^ ^]^ Additionally, this protein is recognized as a significant risk factor for atherosclerotic stroke involving large arteries.^[^ ^48^ ^]^ S100A8, together with S100A9, forms calcium‐binding protein heterodimers involved in vascular inflammation and leukocyte recruitment.^[^ ^25^ ^]^ S100A9 has been detected in thrombi and is strongly correlated with stroke‐related mortality.^[^ ^49^ ^]^ From a translational perspective, our findings suggest that targeting migrasome biogenesis, trafficking, or the release of their bioactive cargo may offer novel therapeutic strategies to mitigate early inflammatory injury following stroke. Given the relative accessibility of peripheral blood, migrasome‐derived proteins such as SERPINA1 and S100A8 also hold promise as diagnostic or prognostic biomarkers.

Despite these insights, our study has several limitations. First, we relied on peripheral blood samples from IS patients due to the inaccessibility of clinical brain tissue, which constrained our ability to directly examine lesion‐specific features in human subjects. As a result, much of our validation relied on experimental animal models. Second, although our multi‐omics analysis revealed metabolic disturbances involving cholesterol synthesis and endocrine‐related pathways in IS patients, the integrative analysis of proteomic and metabolomic data remains preliminary. Multi‐omics approaches inherently involve high complexity, and more comprehensive, mechanistically integrated analyses are warranted. Finally, while our clinical sample was drawn from multiple centers, it consisted exclusively of Chinese patients, limiting generalizability. Future studies in more diverse populations are needed to validate and expand upon our findings.

## 4 Conclusion

In summary, migrasomes appear to play a crucial role in initiating peripheral inflammation during the early stages of stroke and may contribute to the subsequent development of systemic inflammation and multi‐organ injury. These extracellular vesicles, released by microglia, endothelial cells, and neutrophils, are implicated in complement and coagulation cascades, cholesterol metabolism, and immune modulation following cerebral ischemia. By releasing bioactive substances, migrasomes may influence disease progression and exacerbate injury. Investigating the cellular infiltration processes and the molecular cargo of migrasomes offers valuable insights into stroke pathophysiology and may uncover novel targets for therapeutic intervention. Nevertheless, further large‐scale, multicenter studies are needed to validate these findings and explore their translational potential.

## 5 Experimental Section

### Participant Recruitment and Study Design

This study enrolled a total of 143 participants aged between 30 and 90 years, including 123 patients diagnosed with IS and 20 healthy controls. Participants were recruited from nine centers between August 2018 and October 2020. The study was conducted in accordance with the principles of the Declaration of Helsinki and complied with relevant clinical trial regulations in China. The protocol was registered with the Chinese Clinical Trial Registry (registration number: ChiCTR1800015189). All participants provided written informed consent prior to enrollment. Baseline clinical characteristics are detailed in Table 1 and Figure S1 (Supporting Information). For subsequent migrasome isolation, peripheral blood samples from three randomly selected participants (within the above‐registered cohort) were additionally processed under the ethics approval granted by the Ethics Committee of the First Affiliated Hospital of Zhejiang Chinese Medical University (approval No. 2025‐KLS‐671‐01).

Participants were stratified into groups based on their National Institutes of Health Stroke Scale (NIHSS) scores to classify disease severity: mild (NIHSS ≤ 4), mild‐moderate (5 ≤ NIHSS ≤ 9), and moderate (10 ≤ NIHSS ≤ 15). Additionally, the disease course was categorized by onset time as Process 1 (≤ 7 days), Process 2 (8–13 days), and Process 3 (15–30 days).

### Inclusion Criteria

Eligible participants were those diagnosed with IS according to established Western medical criteria, aged between 30 and 90 years, experiencing either their first stroke or having a history of stroke without residual disability at the time of the current episode. All subjects voluntarily consented to participate and signed informed consent documents. Anterior circulation infarction was confirmed via imaging modalities (computed tomography [CT] or magnetic resonance imaging [MRI]) and classified as total or partial anterior circulation infarction according to the Oxfordshire Community Stroke Project (OCSP) clinical classification. Symptom onset was defined as occurring within 1 month prior to enrollment. NIHSS scores ranged from above 4 to below 20. There were no restrictions based on sex.

### Participant Exclusion Criteria

The following exclusion criteria were applied: Patients with bleeding disorders or a history of bleeding within the 3 months preceding enrollment; patients diagnosed with post‐stroke depression or dementia; individuals with cerebral hemorrhage following ischemic stroke; those with posterior circulation infarction (POCI), transient ischemic attack (TIA), intracranial vascular malformations, asymptomatic cerebral infarctions, large‐area cerebral infarctions, or unstable vital signs. Patients who had discontinued herbal treatments for IS less than 1 week prior to or had received thrombolytic therapy after the current onset were excluded. Additionally, patients with comorbidities affecting limb motor function—such as lameness, osteoarthritis, rheumatoid arthritis, or gouty arthritis—that could interfere with neurological assessments were excluded.

Patients with hemiplegia resulting from brain tumors, traumatic brain injury, cerebral cysticercosis, or cerebral embolism secondary to rheumatic heart disease, coronary heart disease, or other cardiac conditions were excluded. Those with severe impairment in daily activities due to various diseases or preexisting physical debilitation before the current illness were not eligible. Patients with severe cardiovascular, hepatic, renal, or hematopoietic disorders, or those who had undergone major surgery recently, were excluded.

Further exclusions included individuals with psychiatric disorders, severe depression, alcohol dependence, or a history of substance abuse; pregnant or nursing women, or those planning to conceive within three months after discontinuing study participation; patients with known allergic predispositions, multiple drug allergies, or allergies to components of the study medication; participants involved in other clinical trials within three months prior to enrollment; and any other individuals deemed unsuitable for participation by the investigators.

### Protein Extraction and MS Assay for DIA

A total of 2 µL of plasma samples were mixed with 98 µL of 50 mm ammonium bicarbonate (ABC) buffer, followed by heat inactivation at 95 °C for 3 min. Samples were then digested overnight at 37 °C with trypsin at an enzyme‐to‐protein ratio of 1:25. The resulting peptides were collected and dried using a SpeedVac system (Eppendorf).

For proteome profiling, the dried peptides were reconstituted and loaded onto a 2‐cm self‐packed trap column (100 µm inner diameter, packed with 3 µm ReproSil‐Pur C18‐AQ beads, Dr. Maisch GmbH) using solvent A (0.1% formic acid in water). Peptide separation was performed on a 15‐cm‐long analytical column with a 150 µm inner diameter, packed with 1.9 µm ReproSil‐Pur C18‐AQ beads (Dr. Maisch GmbH). Following electrospray ionization at 2 kV, peptides were introduced into a Q Exactive HF‐X Hybrid Quadrupole‐Orbitrap Mass Spectrometer (Thermo Fisher Scientific) coupled with an EASY nLC 1200 high‐performance liquid chromatography system (Thermo Fisher Scientific). Mass spectrometry data acquisition employed a data‐independent acquisition (DIA) strategy. The MS1 scan ranged from 300 to 1400 m/z at a resolution of 60 000 with an Automatic Gain Control (AGC) target of 4e5 or a maximum injection time of 50 ms. This was followed by 30 DIA segments acquired at 15 000 resolution with an AGC target of 5e4 or 22 ms maximum injection time. High‐energy collision dissociation (HCD) fragmentation was conducted with a normalized collision energy set to 27%. Spectra were collected in profile mode, with the default charge state for MS2 set to 3.

Raw data files were processed and analyzed for both identification and quantification using the iProteo one‐stop data analysis cloud platform. Quality control measures showed that the mass deviation for identified peptides was predominantly within 10 parts per million (ppm), indicating high accuracy and reliability. The Mascot search engine was employed for spectral library analysis, and peptide score distributions confirmed the high quality of the MS data. Peptides were filtered using a false discovery rate (FDR) threshold of ≤0.05 in the qualitative analysis of DIA‐generated data.

### Metabolite Extraction and LC‐MS/MS Analysis

Plasma samples (50 µL), previously thawed slowly at 4 °C, were transferred into 1.5 mL centrifuge tubes. To each tube, 300 µL of pre‐chilled methanol/water solution (2:1, v/v) was added, followed by vortexing for 30 s to ensure thorough homogenization. Subsequently, 600 µL of methyl tert‐butyl ether (MTBE) was added, and the mixture was vortexed for an additional 30 s. The tubes were then immersed in an ice‐cold bath and subjected to ultrasonication for 10 min to facilitate metabolite extraction. Following ultrasonication, samples were centrifuged at 14 000 × g for 10 min at 4 °C, resulting in phase separation into an upper lipid‐containing layer and a lower aqueous phase enriched with small‐molecule metabolites. Both layers were carefully collected into separate Eppendorf tubes, then vacuum‐dried at room temperature and stored at −80 °C until further LC‐MS/MS analysis.

### LC‐MS/MS Analysis of Small Molecule Metabolites

Sample separation was performed using a Nexera LC‐30A microflow ultra‐high‐performance liquid chromatography (UHPLC) system. Prior to sample injection, the chromatographic column was equilibrated with 98% mobile phase A. Samples were introduced via an autosampler at a flow rate of 0.3 mL min^−1^ onto a Waters ACQUITY UPLC BEH Amide HILIC column (1.7 µm particle size, 2.1 × 100 mm). Gradient elution was conducted by increasing the proportion of mobile phase B from 2% to 98% over 11.5 min. The gradient profile was as follows: starting with 2% mobile phase B maintained for 0.5 min, followed by a 0.1 min ramp to 98% mobile phase B, which was held for 4 min, then a 1.9 min column cleaning step at 98% mobile phase B before returning to the initial conditions. Chromatographically separated compounds were analyzed using a Q Exactive HF‐X mass spectrometer operating in both positive and negative ion modes. Full MS1 scans were acquired over a mass‐to‐charge (m/z) range of 70–1050 with a resolution of 120 000, an automatic gain control (AGC) target of 3 × 10^6^, and a maximum ion injection time of 100 ms. MS2 fragmentation scans were performed at a resolution of 7500 with an AGC target of 2 × 10^5^ and a maximum injection time of 50 ms. High‐energy collision dissociation (HCD) fragmentation was conducted at normalized collision energies of 20, 40, and 60, with product ions scanned within the m/z range of 200–2000 using an isolation window of 1.5 m/z.

### LC‐MS/MS Analysis of Lipids

Sample separation was performed using a Nexera LC‐30A microflow ultra‐high‐performance liquid chromatography (UHPLC) system. The chromatographic column was initially equilibrated with 98% mobile phase A. Samples were then injected via an autosampler at a flow rate of 0.26 mL min^−1^ onto a Waters ACQUITY UPLC BEH Amide HILIC column (1.7 µm particle size, 2.1 × 100 mm). Gradient elution was carried out by a linear increase of mobile phase B from 30% to 100% over 5 min. The gradient started at 30% mobile phase B and was maintained for 20 min, followed by a brief 0.1 min adjustment back to 30% mobile phase B. A 2.9 min column cleaning step at 100% mobile phase B was performed after 5 min of operation at this condition. Chromatographically separated lipids were analyzed using a Q Exactive HF‐X mass spectrometer in both positive and negative ion detection modes. Full MS1 scans were acquired over an m/z range of 200–2000 at a resolution of 120 000, with an automatic gain control (AGC) target of 1 × 10^6^ and a maximum ion injection time of 100 ms. MS2 fragmentation scans were performed at a resolution of 15 000, with an AGC target of 2 × 10^5^ and a maximum injection time of 80 ms. Fragmentation was achieved using high‐energy collision dissociation (HCD) at normalized collision energies of 20, 40, and 60. Product ions were scanned within an m/z range of 200–2000 with an isolation window of 1.5 m/z. Raw data files (RAW format) obtained from the MS analysis were processed using Progenesis QI software, which facilitated database searching and compound identification.

### Data Processing

Missing values were addressed using a matching runs strategy, a common and effective imputation approach in proteomics and related fields.^[^ ^50^, ^51^ ^]^ A dynamic regression model was applied to track peptide sequences across samples. This model estimated the retention times (RTs) of putative peptides through linear or quadratic functions depending on the correlation coefficient (R^2^). The predicted RTs were validated by analyzing the extracted ion chromatograms corresponding to the expected m/z values.^[^ ^52^, ^53^ ^]^

### Differential Analysis and Pathway Enrichment Analysis

Fold changes (FC) of average protein or metabolite abundances between groups were calculated using log2 transformation. The Wilcoxon signed‐rank test was applied for comparisons between two groups, while the Kruskal–Wallis test was employed for comparisons involving three or more groups. Enrichment scores for individual samples of differentially expressed molecules were computed using gene sets derived from the KEGG database^[^ ^54^ ^]^ and Reactome pathways.^[^ ^55^ ^]^ False discovery rate (FDR) adjustment was performed using the Benjamini–Hochberg method, with FDR < 0.05 considered statistically significant.

To predict tissue damage severity related to IS, proteomic data were mapped to cell‐type signature gene modules obtained from the MSigDB database.^[^ ^56^ ^]^ Cell types were scored using Gene Set Variation Analysis (GSVA),^[^ ^57^ ^]^ and differential cell‐type enrichments were visualized. Considering the specificity of IS samples, distinct tissue‐resident cells were selectively highlighted, focusing on primary organs including brain, heart, lung, liver, stomach, spleen, and kidneys.

Peripheral immune cell characteristics of IS patients were further analyzed using the xCell method (https://xcell.ucsf.edu).^[^ ^58^ ^]^ GSVA was applied to score proteomic data from both control and IS groups to reveal immune landscape differences.

### PCA

Principal Component Analysis (PCA) was conducted using R (version 4.2.2) on a dataset consisting of 7725 proteins, 3767 lipids, and 2125 small molecules extracted from 143 plasma samples. This analysis aimed to elucidate molecular differences in peripheral blood among participants, including comparisons between control subjects and IS patients, across varying IS severity groups, and among different infarct locations.

### Consensus Clustering Analysis

Consensus clustering analysis was performed on the protein expression matrix using the ConsensusClusterPlus R package (version 1.46.0).^[^ ^59^ ^]^ The analysis involved testing a range of cluster numbers (k) up to a maximum of 10. In each iteration, 80% of the samples were resampled over 1000 iterations. Hierarchical clustering was performed using the k‐means (km) algorithm, with Euclidean distance as the similarity metric. Optimal cluster separation was observed at k = 3, which showed significant correlation with participants’ clinical characteristics.

Weighted Gene Co‐expression Network Analysis (WGCNA) was conducted using the WGCNA R package (version 1.72‐1).^[^ ^60^ ^]^ Initially, the soft‐thresholding power was determined via the softConnectivity and pickSoftThreshold functions. A power less than 30 that achieved a scale‐free topology fit index of 0.9 and maintained adequate network connectivity was selected to construct the scale‐free network. Using this power, the adjacency matrix was computed and subsequently transformed into a topological overlap matrix (TOM). Gene modules were identified using the dynamic tree‐cut algorithm with parameters set to a minimum module size of 30 and a deep split of 2. Modules were further refined using hierarchical clustering and dynamic merging with a cut height of 0.25. Correlations between module eigengenes and feature genes were calculated using Pearson's correlation coefficient. Modules with a correlation coefficient (r) ≥ 0.9 and *p* < 0.05 were considered statistically significant.

### Mice

Healthy male C57BL/6 mice (25 ± 5 g) were purchased from Zhejiang University of Traditional Chinese Medicine. C57BL/6J *Tspan9^‒/‒^* mice were kindly provided by Dr. Li Yu from Tsinghua University. All mice were housed in the Laboratory Animal Research Centre of Zhejiang University of Traditional Chinese Medicine under specific pathogen‐free conditions, with controlled temperature (19 ± 2 °C), relative humidity (50 ± 2%), and a 12 h light/dark cycle. Standard feed and water were provided ad libitum.

### CIRI Model

Mice were anesthetized with intramuscular atropine administered for 10 min, followed by intraperitoneal injection of sulphadoxine. The mice were placed in the supine position, and the mid‐neck area was shaved and sterilized. The common carotid artery was carefully isolated and ligated at its inferior portion. The internal and external carotid arteries were then separated, with the external carotid artery being ligated. A small incision was made between the common and internal carotid arteries to allow insertion of an A4 mouse monofilament wire, which was advanced to occlude the middle cerebral artery for either 1 or 2 h. After the ischemic period, the filament was withdrawn to allow reperfusion. Neurological testing and sample collection were conducted one day following reperfusion.

### Cell Lines

This study utilized two cell types: the human microglia cell line HMC3 and the mouse brain microvascular endothelial cell line bEnd.3. Both cell lines were cultured in DMEM medium (Gibco, Cat. No. 11965118) supplemented with fetal bovine serum (Gibco, Cat. No. A5669401) and 100 U mL^−1^ penicillin‐streptomycin (Gibco, Cat. No. 15140122) to promote growth. Cells were maintained at 37 °C in a humidified incubator with an atmosphere of 95% air and 5% CO_2_.

### Oxygen‐Glucose Deprivation/Reoxygenation (OGD/R) Model

To simulate ischemic conditions, cells were incubated in glucose‐free DMEM medium (Gibco, Cat. No. 11966025) under hypoxic conditions (5% CO_2_, 94% N_2_, 1% O_2_) for 3 h. This was followed by reoxygenation with normal DMEM medium under normoxic conditions for 21 h.

### Extraction and Purification of Peripheral Blood Immune Cell‐Derived Migrasomes

Peripheral blood migrasomes derived from whole immune cells were isolated using the MigraIso FULL Pro kit (MGS‐P007, Migrasome Therapeutics) following the manufacturer's instructions. The key steps are as follows: 1) Whole blood was collected in EDTA anticoagulation tubes and centrifuged to separate plasma. 2) Optimizers, enhancers, and promoters were added to reduce migrasome adherence and loss during processing. 3) The plasma was diluted with a diluent and centrifuged at 20 000 × g at 4 °C to obtain a crude migrasome pellet. 4) The pellet was resuspended and incubated with a cocktail of negatively selected antibodies, then centrifuged again at 20 000 × g to remove unbound antibodies. 5) After resuspension, magnetic beads were added and incubated to remove vesicles originating from platelets, erythrocytes, epithelial cells, and other non‐immune cell sources through magnetic separation. 6) The remaining solution was centrifuged at 20 000 × g to isolate migrasomes enriched from various immune cell origins.

### Measurement of Cerebral Blood Flow

Mice were anesthetized according to the established protocol and positioned prone. The scalp was shaved and disinfected, followed by a “T”‐shaped incision to expose the skull. The exposed skull was irrigated with saline. Bilateral cerebral blood flow was then measured using laser speckle contrast imaging (RWD Life Science Co., Ltd., China). The resulting images were processed to obtain the final cerebral blood flow measurements.

### Extraction and Purification of Mouse Brain Migrasomes

The right hemisphere of the mouse brain was mechanically sheared and then digested for 20 min with papain (20 U mL^−1^; BS190‐25 g, Biosharp) and DNase I (150 U mL^−1^; D5025, Keruihui Technology).^[^ ^61^ ^]^ The digested mixture was centrifuged at 1000 × g for 5 min at 4 °C to remove cell bodies, and the supernatant was collected. The remaining pellet was subjected to a second digestion with the same enzyme mix for an additional 20 min, followed by centrifugation again at 1000 × g for 5 min at 4 °C to collect the supernatant. The combined supernatants were centrifuged at 20 000 × g for 20 min at 4 °C to pellet the crude migrasomes. This pellet was then subjected to gradient separation using an Optiprep kit (Sigma–Aldrich).^[^ ^62^ ^]^ Migrasomes were collected from the third to fifth gradient layers for subsequent identification and analysis.

### Flow Cytometry

One day after reperfusion, blood was collected via cardiac puncture into EDTA anticoagulant tubes. The liver, lungs, and kidneys were mechanically dissociated and then digested with a solution containing 0.1% collagenase IV (WBC‐LS004186, Worthington), 0.01% DNase I (Worthington), and HBSS, followed by incubation at 37 °C for 30 min. The digested tissue was filtered through a 100 µm nylon mesh to obtain a single‐cell suspension.^[^ ^63^ ^]^ Spleens were mechanically ground using a mortar and pestle to generate single‐cell suspensions. For blood, liver, lung, kidney, and spleen samples, erythrocytes were lysed by adding five volumes of erythrocyte lysis buffer (C3702‐120 mL; Bicentennial) and incubating for 3 min. The cells were then centrifuged at 300 × g for 5 min in PBS to collect the pellet.

For the heart, single‐cell suspensions were obtained by mechanical dissociation and enzymatic digestion using 0.1% collagenase II (WBC‐LS004174, Worthington), DNase I at 0.6 mg mL^−1^, and HBSS, incubated at 37 °C for 30 min.^[^ ^64^ ^]^ Brain single‐cell suspensions were prepared as previously described.

For flow cytometry staining, 1 × 10^6^ cells were resuspended in 200 µL PBS, then incubated with antibodies against Tspan4 (MBS9461955; MyBioSource) and Ly6g (560602; BD Pharmingen) in the dark for 20 min. After staining, 1 mL PBS was added, and cells were centrifuged at 300 × g for 5 min. The pellet was resuspended in 200 µL PBS and analyzed using a CytoFLEX S flow cytometer (Beckman Coulter). Data were processed and analyzed using FlowJo software version 10.10.0.

### Quantitative Analysis of Peripheral Blood Total Immune Cells and Neutrophil‐Derived Migrasomes

Peripheral blood samples were collected from mice using EDTA as an anticoagulant. Plasma was separated from the blood samples. The blood was diluted to a final volume of 1 mL and centrifuged at 20 000 × g for 1 h to isolate crude migrasomes. The pellet was resuspended and stained with antibodies against CD45, Ly6g, and Apotracker at 4 °C. Migrasomes were visualized using a Dragonfly confocal microscope (Nikon A1), and particle quantification was performed using Imaris software version 10.0^16^.

### Hematoxylin, Eosin (H&E) Staining and Multiplex Immunohistochemistry (mIHC)

Mouse brains were subjected to gradient dehydration, paraffin embedding, and sectioned into 4 µm slices mounted on slides. The slides were oven‐dried at 60 °C for 2 h. H&E staining was conducted using an automated staining machine. After staining, the slides were dried again at 60 °C for 15 min and sealed.

For mIHC, endogenous peroxidase activity was quenched by incubation with 3% hydrogen peroxide following antigen retrieval with sodium citrate buffer. Non‐specific binding was blocked using 3% bovine serum albumin (BSA). Primary antibodies against CD31 (1:500, ab182981, Abcam) and Iba1 (1:500, ab283319, Abcam) were incubated overnight at 4 °C. The next day, slides were incubated with goat anti‐rabbit/mouse horseradish peroxidase (HRP)‐conjugated secondary antibody (Aifang Biotechnology) for 50 min. Tyramide signal amplification was performed by incubating the slides with TSA dye (488 Tyramide, Share‐bio) for 3 min. Antibody stripping was achieved by microwave treatment, followed by blocking with 3% BSA and overnight incubation at 4 °C with a primary antibody against Tspan4 (MBS9461955; MyBioSource). Finally, slides were mounted using a DAPI‐containing mounting medium (BL739B; Biosharp). Images were captured during the drying process and analyzed using ImageJ software version 1.6.0_20.^[^ ^65^ ^]^

### Electron Transmission Microscopy (TEM) of Isolated Migrasomes

Isolated and purified migrasomes were adsorbed onto copper grids and negatively stained with phosphotungstic acid for 30 s. After adsorption and drying, the samples were examined using a transmission electron microscope.

### Scanning Electron Microscopy (SEM)

Cells were cultured in fibronectin‐coated Petri dishes (F8180, Solarbio) at room temperature and then subjected to the oxygen‐glucose deprivation/reoxygenation (OGD/R) model. Following treatment, cells were fixed in 2.5% glutaraldehyde solution at 4 °C for 16 h. After fixation, samples were rinsed twice with distilled water for 10 min each.

Cells were dehydrated sequentially in graded ethanol solutions (50%, 70%, 80%, 90%, and 100%), followed by graded tert‐butyl alcohol concentrations (50% to 100%), with each step lasting 10 min. The samples were then dried using a low‐temperature vacuum drying method with tert‐butyl alcohol. Finally, the prepared samples were examined under a scanning electron microscope.

### Cytokine Analysis of Plasma Using Multiplex Bead‐Based Immunoassay

Mouse plasma cytokine levels were measured using the Bio‐Plex Pro Mouse Cytokine Group 1 Panel 23‐Plex (M60009RDPD/64635111, Bio‐Rad). Whole blood was collected from mice into EDTA anticoagulant tubes and centrifuged at 2000 × g for 10 min at 4 °C to separate plasma. The supernatant was then further centrifuged at 12 000–16 000 × g for 10 min at 4 °C to obtain the upper plasma layer. Plasma samples were diluted 1:2 by shaking and centrifugation. Subsequently, 50 µL of each diluted sample was analyzed according to the manufacturer's instructions.

### Animal Ethics

All animal experiments were conducted following the Principles of Laboratory Animal Care and were approved by the Animal Ethics Committee of Zhejiang University of Traditional Chinese Medicine, Hangzhou, China (approval numbers: IACUC‐20240909‐03, IACUC‐20250603‐14).

### Statistical Analysis

Statistical analyses were performed using SPSS version 25.0 (IBM Corp., Armonk, NY), R version 4.2.2 (R Foundation for Statistical Computing, Vienna, Austria), and GraphPad Prism 8. Specific statistical methods are detailed in figure legends. Group comparisons for continuous variables were conducted using the Wilcoxon signed‐rank test, t‐test, Mann–Whitney U test, or Kruskal–Wallis test as appropriate. For multiple‐group comparisons, Tukey's multiple comparison test or Dunnett's t‐test was applied. Categorical variables were analyzed using χ^2^ or Fisher's exact tests. Data are presented as means ± standard errors of the mean (SEM) for normally distributed variables with homogeneity of variance, and as medians with interquartile ranges for non‐normally distributed variables. Categorical data are expressed as frequencies (%). Statistical significance was set at *p* < 0.05. Significance annotations are as follows: ^****^ *p* < 0.0001, ^***^ *p* < 0.001, ^**^ *p* < 0.01, ^*^ *p* < 0.05 compared with control groups.

## Conflict of Interest

The authors declare no conflict of interest.

## Author Contributions

H.Z., Y.Z., P.Z., H.W., and L.L. contributed equally to this work. H.Z., YZ., P.Z., H.W., and L.L. contributed to the study concept and design. H.Z., Y.Z., P.Z., and L.L. were responsible for the data acquisition and analysis. H.Z., Y.Z., P.Z., and L.L. wrote the manuscript. B.X., Y.W., Y.S., Y.K., H.Z., M.S., and Q.H. contributed to the collection of samples and clinical data, J.Y., C.D., W.F., B.Z., and H.W. contributed to the critical revision of the manuscript. All authors revised and approved the final version.

## Supporting information



Supporting Information

## Data Availability

All data from this study are presented in the paper and/or supplementary material. Original proteomic data were deposited in iProX IPX0007470000 (https://www.iprox.cn/page/home.html). Raw metabolomic data were stored in MetaboLights with entry number MTBLS8873 (https://www.ebi.ac.uk/metabolights).
