# Supplementary material for: Migrasomes in Ischemic Stroke: Molecular Landscape and Pathophysiological Impact
Source: Adv Sci (Weinh). 2025 Dec 3;13(6):e20608. doi: 10.1002/advs.202520608 (PMC12866748; doi:10.1002/advs.202520608)
Supplement: Supplementary file 1 — Supporting Information [file ADVS-13-e20608-s001.docx]

Supplementary Materials for

Migrasomes in Ischemic Stroke: Molecular Landscape and Pathophysiological Impact

Huifen Zhou, Yingying Zhang, Peng Zhou, Haofang Wan, Lingling Li, Bin Xu, Yuping Wan, Yingming Song, Yi Kang, Hongbo Zhang, Min Shi, Qun Hou, Jiehong Yang, Chen Ding, Wei Fu, Buchang Zhao, Haitong Wan

Correspondence to: whtong@126.com(H.W.), yjhong@zcmu.edu.cn(J.Y.), chend@fudan.edu.cn(C.D.), fv_fv_fv@163.com(W.F.), naoxintongzhi@163.com(B.Z.)

**This PDF file includes:**

Figure. S1 to S5

Table S1

**Figure S1.**


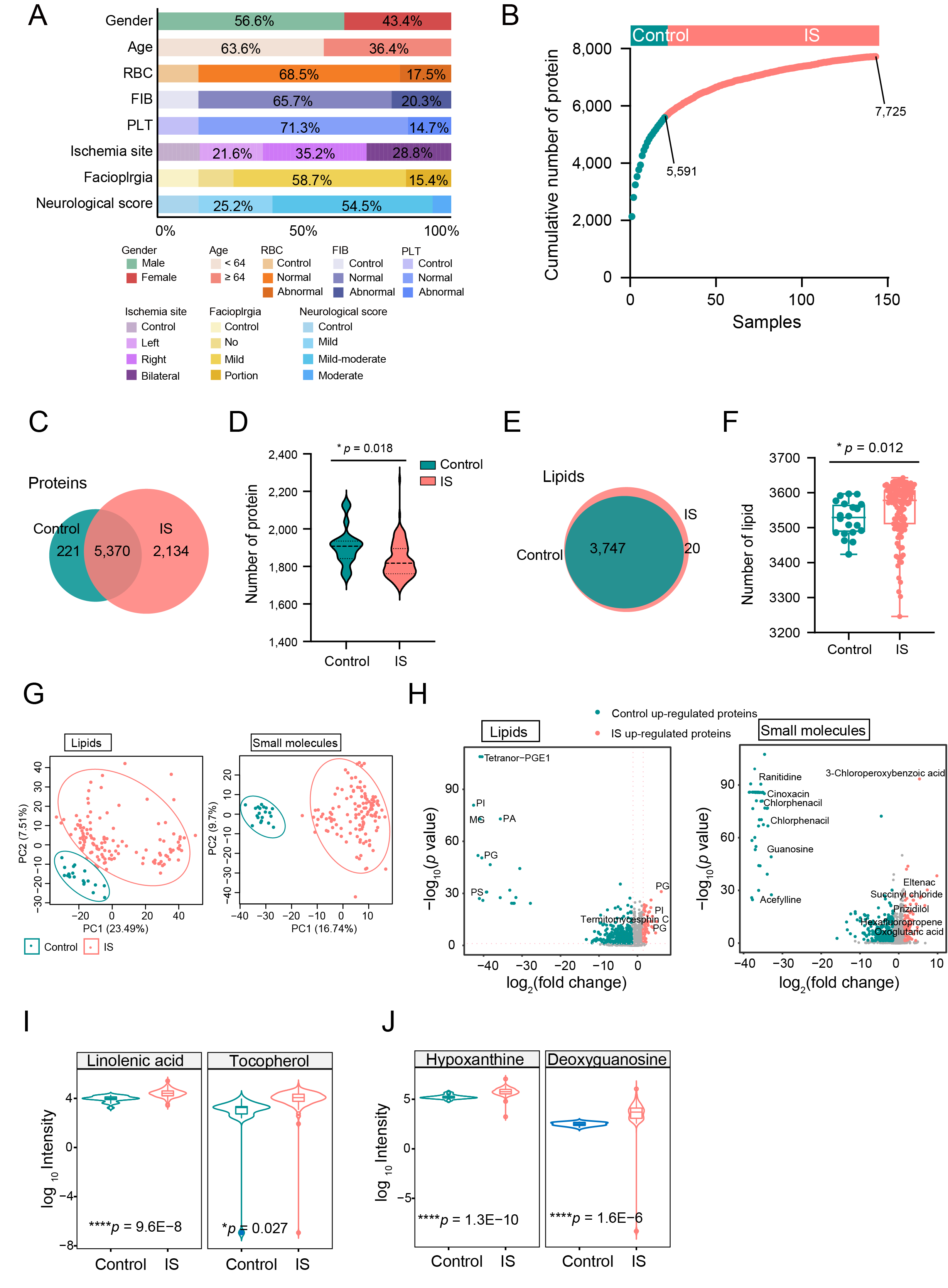


**Figure S1.** Signatures of ischemia stroke from multiomics. (A) Bar chart showing the proportion of all population with different pathological characteristics. (B) Protein expression distribution of 143 samples. (C) Venn plot showing the identification numbers of IS samples was significantly higher than that of the Control group analyzed by Wilcoxon-signed rank test. (D) Violin diagram showing protein identification numbers and overlapping proteins in Control and IS samples. (E) Venn plot showing the numbers of identified lipids in the stroke group samples was significantly higher than that of the Control group analyzed by Wilcoxon-signed rank test. (F) Boxplot chart showing lipid identification numbers and overlapping lipids in Control and IS samples. (G) PCA analysis results showing a clear demarcation between lipid and small molecule metabolites in the Control group and IS group analyzed by Wilcoxon-signed rank test. (H) The volcano plot displaying high expression lipid and small molecule metabolites in the IS group (highlighted in red) and high expression proteins in the Control group (highlighted in green), analyzed by Wilcoxon-signed rank test. (I) Violin plot/boxplot showing a significantly high expression of linolenic acid and tocopherol in the IS group analyzed by Wilcoxon-signed rank test. (J) Violin plot/boxplot showing a significantly high expression of hypoxanthine and deoxyguanosine analyzed by Wilcoxon-signed rank test. Abbreviation: IS, ischemic stroke.

**Figure S2.**


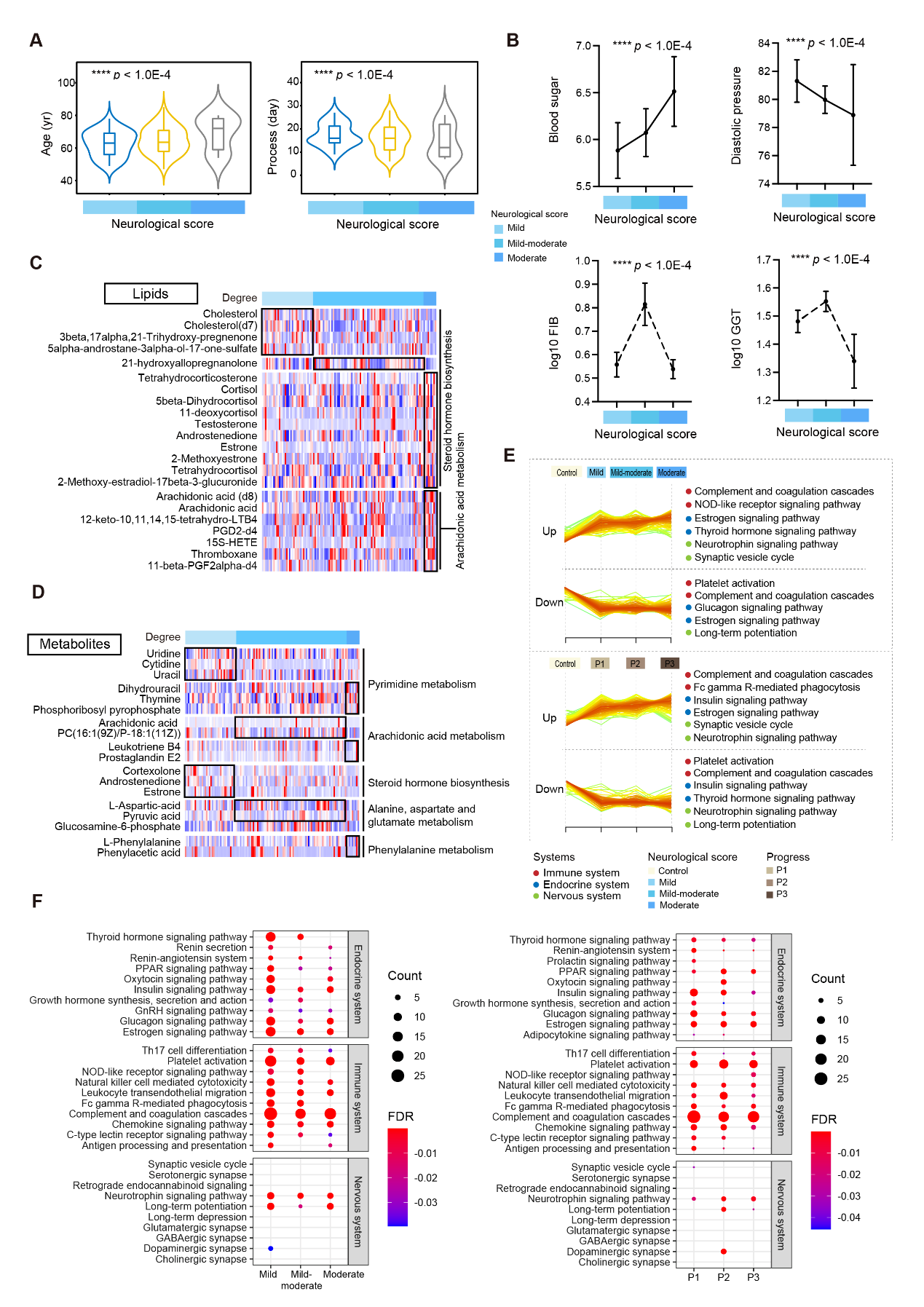


**Figure S2.** Metabolic and clinical characteristics of patients with different stroke severities. (A) The violin plots/box plots indicate that the moderate stroke population tends to have a higher average age, while the mild stroke population exhibits a longer disease duration, as determined by the Fisher’s exact tests. (B) Boxplots showing high blood sugar and low diastolic hypotension in the moderate population, and high expression of FIB and GGT in the mild-moderate population, analyzed using the Fisher’s exact tests. (C) The heatmap shows the primary small-molecule metabolic signals and the heatmap displays the main signals and expression patterns of associated small molecules within the mild-to-moderate IS population. (D) Heatmap presents the main lipid metabolic signals within the mild-moderate IS population and the corresponding expression levels of associated metabolic molecules. (E) Muffz chart showing different proteins with different disease severities/courses in the IS population. (F) Three system-related enrichment pathways. Abbreviation: IS, ischemic stroke.

**Figures S3.**

**
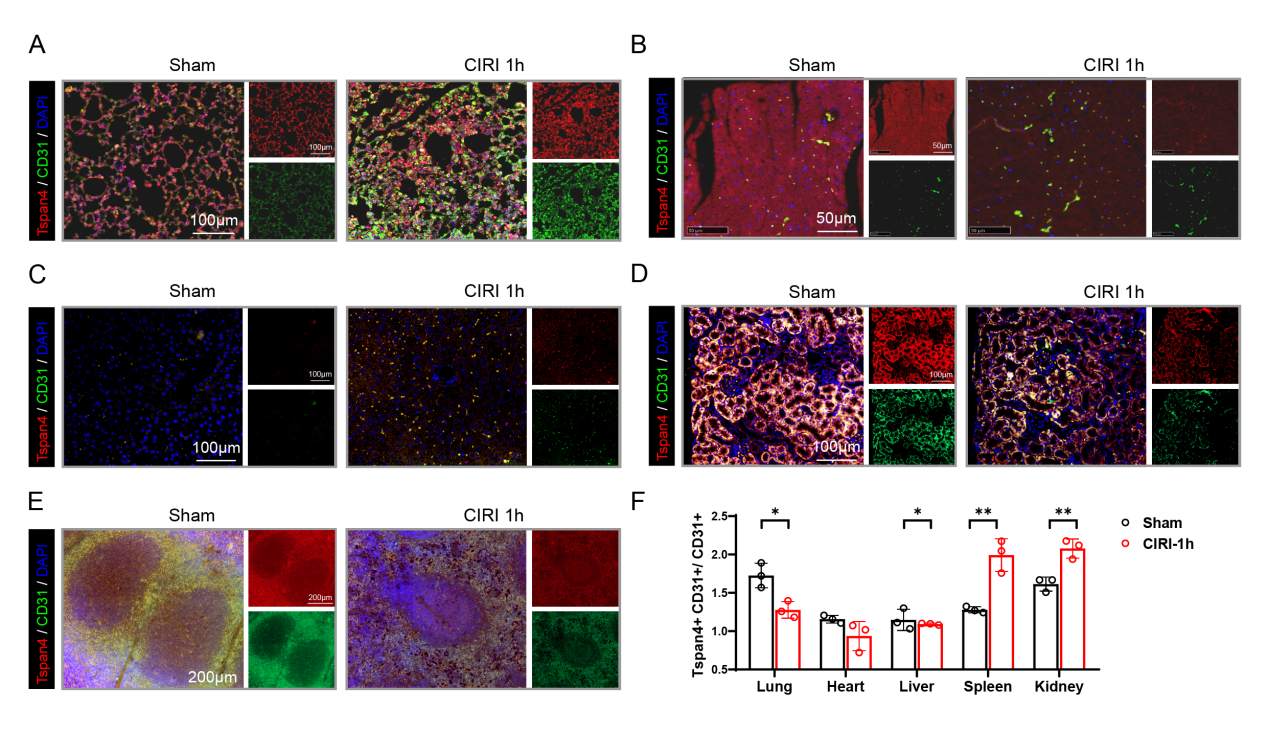
**

Figure S3. Immunofluorescence images of mouse multiple organ Tspan4 and CD31. (A-E) The images demonstrate the fluorescent representative images of tissues from the lungs, hearts, livers, spleens, and kidneys of CIRI-1h experimental stroke mice, respectively. The immunohistochemical colocalization staining utilizes TSPAN4 (red), CD31 (green), and DAPI (blue). (G) Ratio of fluorescence intensity with lung, heart, liver, spleen, and kidney immunostaining for TSPAN4+CD31+/CD31+. Each group n=3, data are expressed as means ± standard deviations (SDs) with indicated significance (Student’s unpaired t test). **P* < 0.05, ***P* < 0.01, ****P* < 0.001, *****P* < 0.0001. Abbreviation: CIRI, cerebral ischemia-reperfusion model.

**Figures S4.**


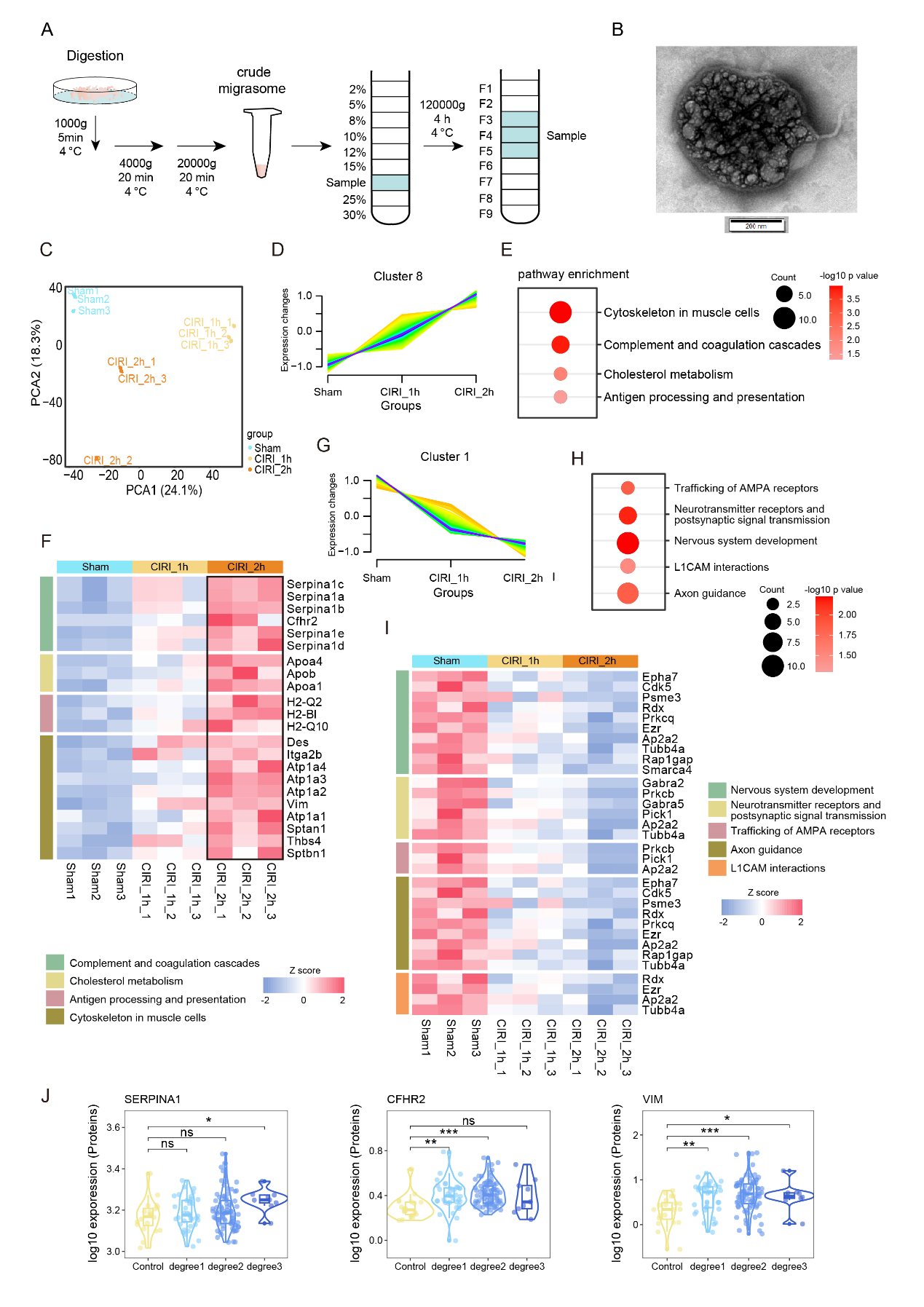


**Figure S4.**  Proteomic characterization of experimental mouse migrasomes. (A) Steps for extracting migrasomes from mouse hemispheres. Combine samples from 6 mice into 1 sample, n=3 per group. (B) Migrasomes identification by electron microscopy. (C) Principal component analysis (PCA) plot illustrating the discrepancy in protein levels between the sham, CIRI-1h, and CIRI-2h groups. (D-E) The k-means analysis demonstrated rising and falling protein trends in the sham /CIRI_1h /CIRI_2h groups. (F-G) Bubble plots showing the signaling pathways for the rising and falling protein trends in the sham/CIRI_1h/CIRI_2h groups. (H-I) Heatmap showing the signaling pathways and pathway molecules for the rising and falling protein trends in the sham /CIRI_1h/CIRI_2h groups. Abbreviation: CIRI, cerebral ischemia-reperfusion model.

**Figure S5.**


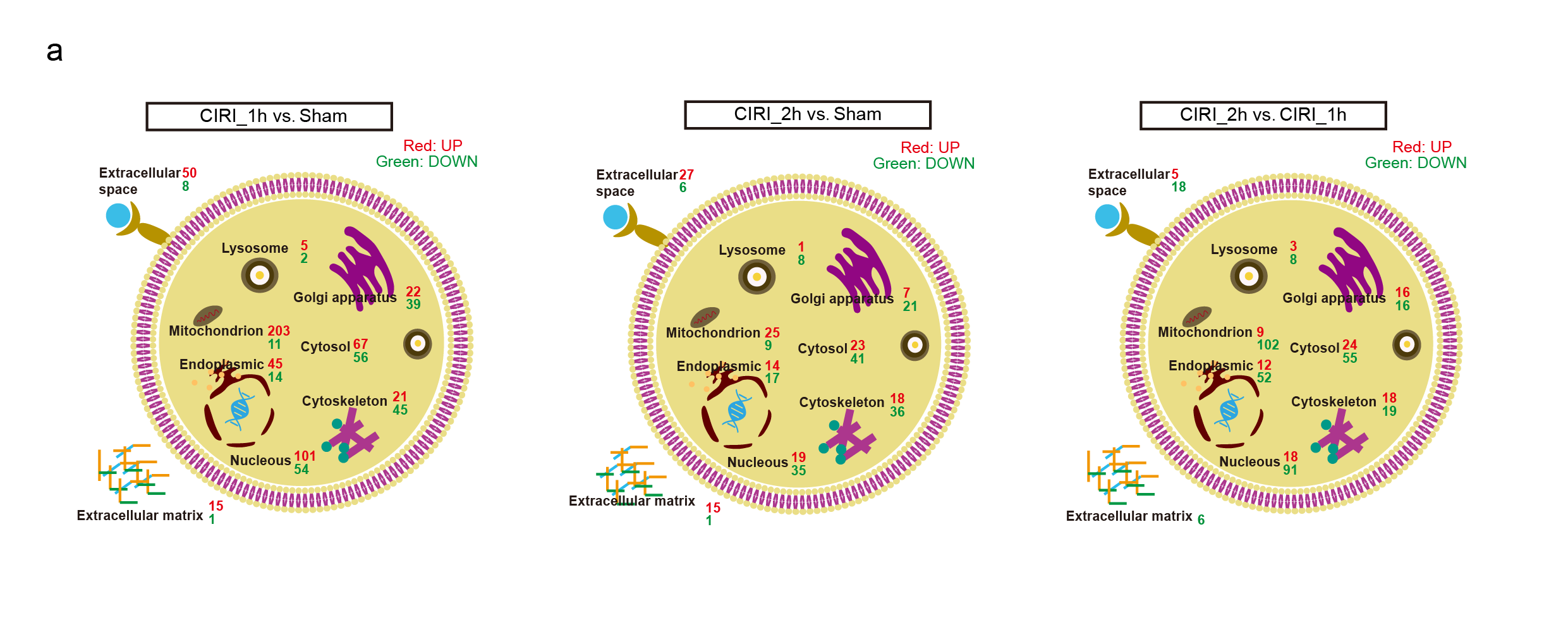


**Figure S5.**  Results of subcellular organelle localization in the migratory bodies of the brains of experimental mice with varying degrees of ischemic severity. The sub-localization analysis demonstrated a trend of organelle-associated protein changes in the CIRI_1h group compared with the sham group, the CIRI_2h group compared with the sham group, and the CIRI_2h group compared with the CIRI_1h group. Abbreviation: CIRI, cerebral ischemia-reperfusion injury.

**Table S1. Baseline characteristics of patients with ischemic stroke by different severity**

|  | **Total**  **(N=123)** | **NIHSS** | | | **P value** |
| --- | --- | --- | --- | --- | --- |
|  |  | **0-4** | **5-9** | **10-15** |  |
| **Age** | 64.40±8.54 | 62.72±7.93 | 64.62±8.42 | 69.22±10.79 | 0.116 |
| **Gender (%)** |  |  |  |  |  |
| **Male** | 81 (65.9) | 22 (27.2) | 54 (66.7) | 5 (6.2) | 0.558 |
| **Female** | 42 (34.1) | 14 (33.3) | 24 (57.1) | 4 (9.5) |  |
| **Progress (Day)** | 63.25±22.31 | 17.39±4.99 | 16.09±5.98 | 14.56±7.31 | 0.342 |
| **Barthel index score** | 16.36±5.82 | 72.36±16.84 | 63.01±21.30 | 28.89±16.54 | **<0.001** |
| **PT** | 11.4 (1.44) | 11.30 (1.19) | 11.81 (1.39) | 12.13 (1.10) | **0.015** |
| **Medication history** |  |  |  |  |  |
| **Hypertension (%)** |  |  |  |  | 0.492 |
| **Yes** | 57 (0.46) | 19 (0.33) | 33 (0.58) | 5 (0.09) |  |
| **No** | 66 (0.54) | 17 (0.26) | 45 (0.68) | 4 (0.06) |  |
| **Diabetes mellitus(%)** |  |  |  |  | 0.930 |
| **Yes** | 22 (0.18) | 6 (0.27) | 14 (0.64) | 2 (0.09) |  |
| **No** | 101 (0.82) | 30 (0.29) | 64 (0.63) | 7 (0.07) |  |
| **Hypertension & Diabetes (%)** |  |  |  |  | 0.474 |
| **Yes** | 13 (0.11) | 3 (0.23) | 8 (0.62) | 2 (0.15) |  |
| **No** | 110 (0.89) | 33 (0.3) | 70 (0.64) | 7 (0.06) |  |
| **Hypercholesterolemia (%)** |  |  |  |  | 0.305 |
| **Yes** | 105 (85.4) | 4 (22.2) | 11 (61.1) | 3 (16.7) |  |
| **No** | 18 (14.6) | 32(30.5) | 67 (63.8) | 6 (5.7) |  |
| **Anticoagulant/antiplatelet therapy (%)** |  |  |  |  | 0.498 |
| **Yes** | 110 (89.4) | 34 (30.9) | 68 (61.8) | 8 (7.3) |  |
| **No** | 13 (10.6) | 2 (15.4) | 10 (76.9) | 1 (7.7) |  |
